# Catalytic conversion of cellulosic biomass to harvest high-valued organic acids

**DOI:** 10.1016/j.isci.2023.107933

**Published:** 2023-09-22

**Authors:** Wubin Yan, Qingqing Guan, Fangming Jin

**Affiliations:** 1 School of Environmental Science & Engineering, Shanghai Jiao Tong University, Shanghai, China; 2 Faculty of Civil Engineering and Mechanics, Kunming University of Science and Technology, Kunming, Yunnan, China

**Keywords:** Physical organic chemistry, Green chemistry, Applied chemistry

## Abstract

Catalytic conversion of biomass provides an alternative way for the production of organic acids from renewable feedstocks. The emerging process contains complex reactions and strategies to cut down those complex biogenic materials into target molecules. Here, we review the catalytic conversion of cellulosic biomass toward high-valued organic acids. This work has summarized the key controlling reactions which lead toward formic acid, glycolic acid, or sugar acids in oxidative conditions and the main pathways for lactic acid or levulinic acid in the anaerobic environment from cellulosic biomass and its derivatives. We evaluate and compare different strategies and methods such as one-pot and two-step conversion. Additionally, the optimization of catalytic reactions has been discussed to realize the design of C–C coupling reactions, the development of multifunctional materials, and new efficient system. In all, this article gives an insight guide to precisely convert cellulosic biomass into target organic acids.

## Graphical abstract

**Figure:**
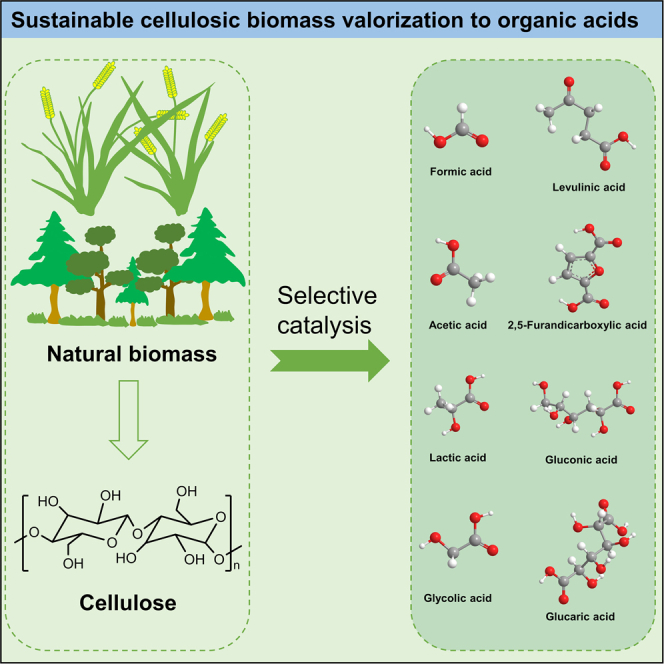

## Introduction

Organic acids are important raw materials in a great demand including leather, chemical synthesis, pharmaceutical synthesis, agriculture, food and other various industries.^1^ Just take formic acid as an example, its global market exceeded US$ 600 million in 2019.^2^ Organic acids used to be produced via selective oxidation of fossil feedstocks. Recently, some researches have also showed the potentials in transforming biomass into acidic products.^3^^,^^4^ Unlike the present petroleum routes to format C−O bonds via the insertion of oxygen atoms, the cleavage of oxygen-enriched natural biomass into organic acids is a sustainable production strategy. Moreover, comparing with the conversion of those oxygen-rich feedstocks into biofuels, it also seems more atom-economic to produce organic acids from biomass due to the avoidance of deoxygenation and some defunctionalization.^5^^,^^6^

Biomass can be defined as biological materials from living or recently living organisms, and a lot of substances belong to biomass.^7^ In the production of organic acids from biomass, cellulosic biomass, which is abundant in forest and agricultural residues, may be the most widely used feedstock due to its aliphatic poly-hydroxyl structures. It was estimated that 4826–7815 million tons of cellulose can be extracted from global major agricultural and forest wastes annually,^8^^,^^9^ which can provide massive feedstocks for the sustainable manufacture of organic acids. However, the selective conversion of cellulosic biomass for desired short-chain organic acid products is challenging. The C-C bonds of cellulose and its derivative molecules can be affected by their adjacent functional groups (e.g., hydroxyl, aldehyde, carbonyl), leading to the difference in the reactivity of various C-C bonds. When the desired C-C bond is cleaved, the bond with lower fracture energy is also broken forming undesired by-products. Besides, the highly hydroxyl-functionlized and hydrogen-bonding characters in cellulose polymer also enhance the difficulties in biomass breaking.^10^

Necessary strategies are required to be taken into consideration to minimize the unwanted byproducts in biomass valorization. The C-C bond which connects an aldehyde group and a hydroxyl/aldehyde group is readily broken under aerobic conditions, in which conditions carbohydrate biomass with aliphatic poly-hydroxyl structure can be used to produce short-chain aliphatic acids.^11^ The catalytic oxidative cleavage of C-C bonds is widely employed to some type of bond scission, but harsh conditions may cause over-oxidation of the intermediates to CO_2_ since the redox environment is hard to be carefully regulated for the oxidative bond cleavage, which have been reviewed.^1^^,^^5^ Meanwhile some researches have showed non-oxidative bond cleavage also plays an equally essential role in the biomass valorization.^6^^,^^12^^,^^13^^,^^14^ A series of processes in the conversion have involved the non-oxidative breaking: hydrolysis of cellulose via the cleavage of glycosidic linkage, C-O bond cleavage via dehydration, C-C bond cleavage via retro-aldol reaction and decarboxylation, and so forth. These non-oxidative bond cleavages can be regulated by acid/base. Additionally, plenty of advanced techniques have been presented and employed to cut the complex polymers for valuable acids. An insight handbook to help understanding the reactions and controlling pathways toward target acids is lacking and necessary.

**Scheme 1:**
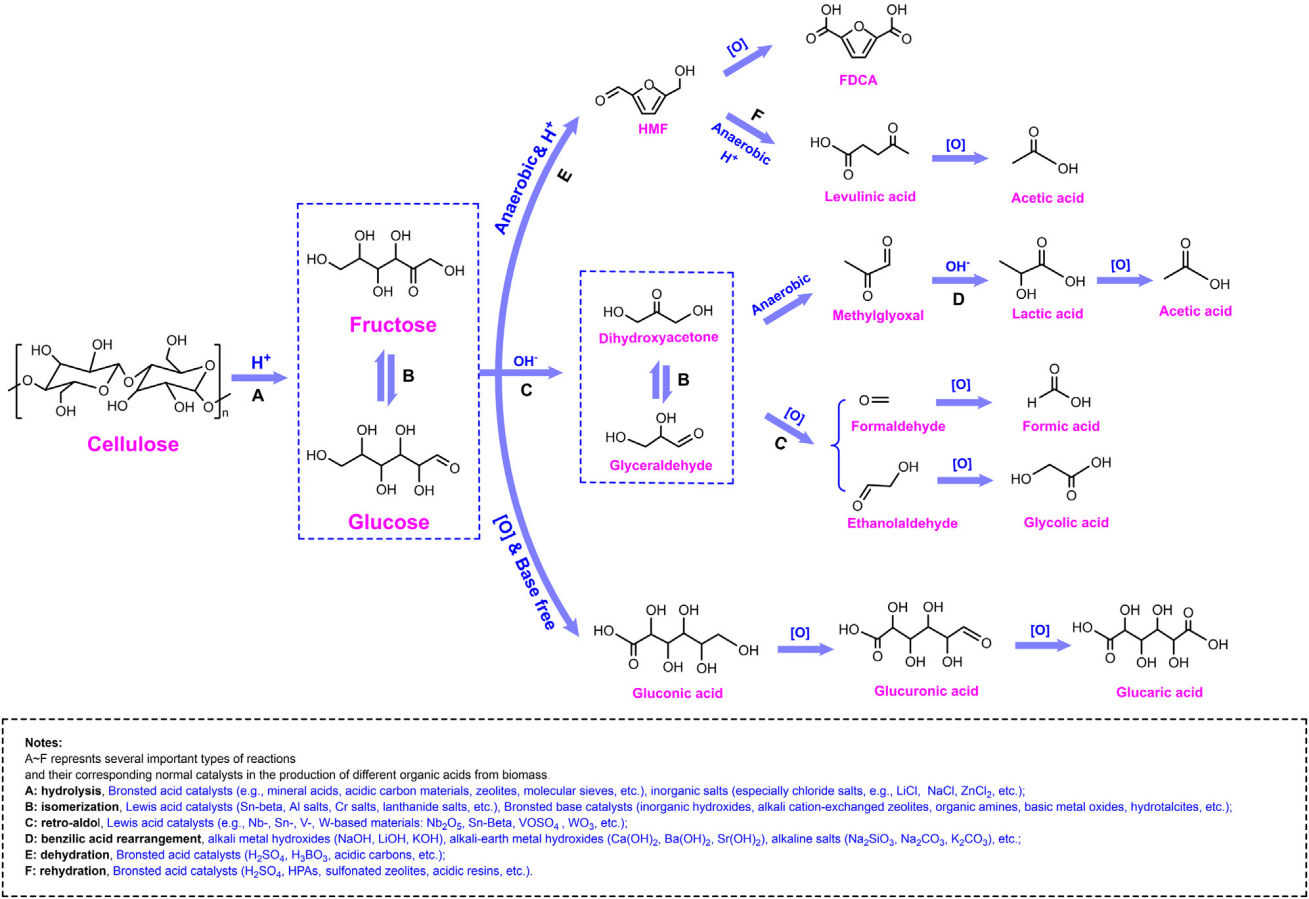
Typical catalytic conversion pathways of cellulosic biomass into value-added organic acids under proper conditions and suitable catalysts

Herein, we concentrate on the catalytic conversion of cellulosic biomass to several typical value-added organic acids: monocarboxylic acids represented by formic acid and acetic acid, hydroxy carboxylic acids represented by lactic acid and glycolic acid, HMF-derived acids presented by 2,5-furandicarboxylic acid (FDCA) and levulinic acid, sugar acids represented by gluconic acid and glucaric acid at the molecular level (Scheme 1). Typical mechanism with new catalysts such as Polyoxometallates (POMs), ionic liquids (ILs), bimetal and co-catalysts are emphatically introduced, aiming at the selective cleavage of target bonds. Additionally, novel strategies including *in situ* extraction and two-step conversion which can exert significant influence on the pathways toward selective biomass breaking have also been described and analyzed. In all, we hope to give a detail guide about catalytic conversion of cellulosic biomass to target high-valued organic acids.

## Oxidative cleavage of cellulosic biomass toward formic and acetic acid

### Oxidation of cellulosic biomass toward formic acid

Formic acid is the simplest organic acid, which is not only a platform chemical widely used in agriculture, rubber, pharmaceuticals, leather, and textile industries, but also a promising hydrogen carrier.^15^ The industrial production of formic acid is via the hydrolysis of methyl formate derived from methanol carboxylation. For carbohydrate biomass, formic acid is produced via oxidative cleavage of the C-C bond which connects an aldehyde group and a hydroxyl/aldehyde group. In a typical conversion of glucose into formic acid, the terminal carbon is oxidized to formic acid and the subsequent C5 intermediate will repeat this degradation until all the C-C bonds are cleaved. In theory, six molecules of formic acid can be obtained. However, the actual bond breaking is challenging to control the selective cleavage of the C-C bonds completely, as by-products can also be produced. CO_2_ is the major by-product from the decarboxylation of acid intermediates.

**Scheme 2:**
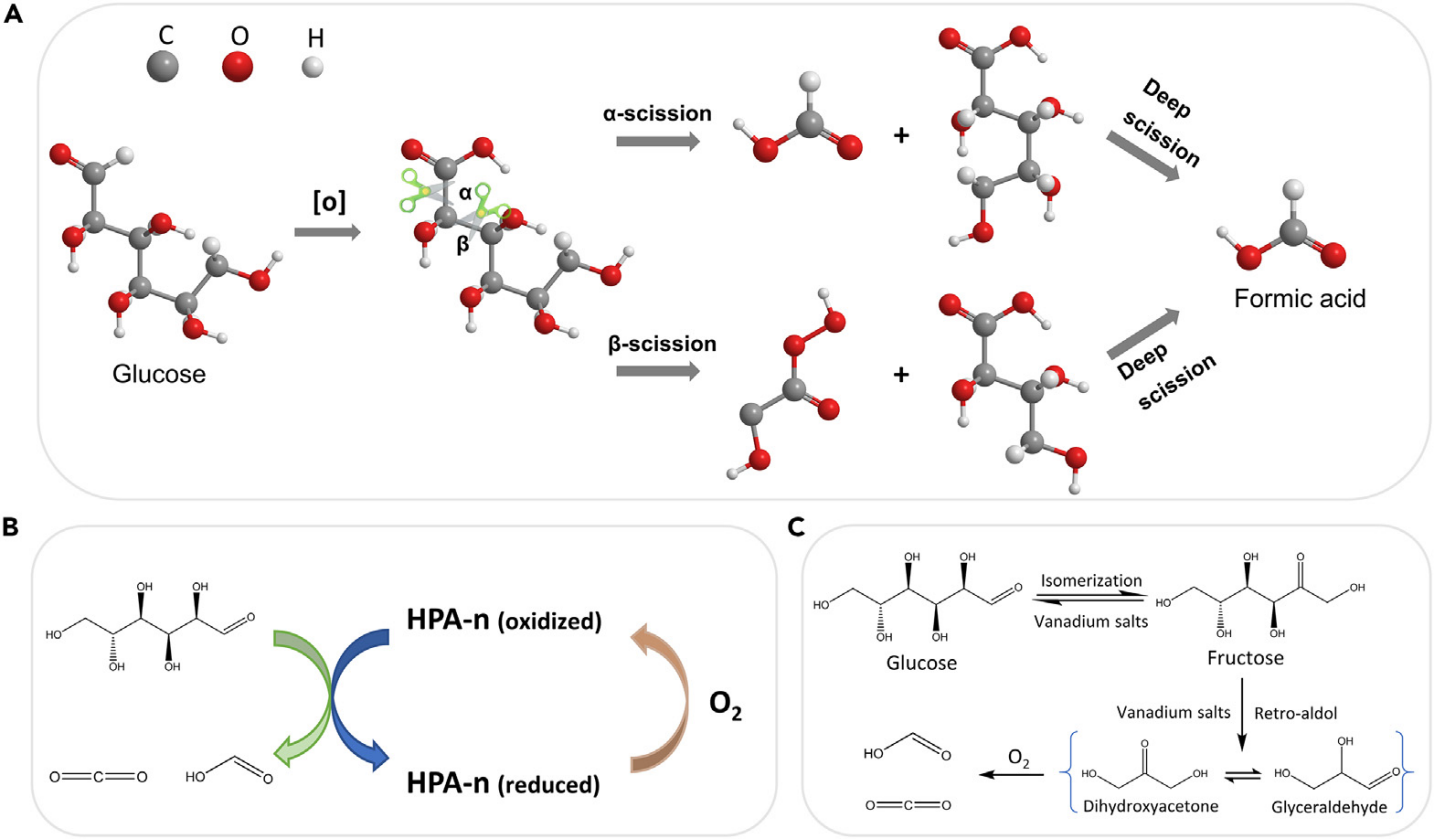
Proposed conversion mechanism of glucose to formic acid (A) α and β C-C bond scission under alkali hydrothermal conditions.^11^ (B) Reaction catalyzed by POMs catalyst.^15^ (C) Pathway in the presence of vanadium salts.^22^

Table 1 shows some typical researches about the biomass oxidation into formic acid, including alkaline hydrothermal oxidation, catalytic oxidation by POMs and vanadium salt catalysts. Keeping the balance between oxidation and over-oxidation is the key point for formic acid production no matter what kind of catalytic system. Usually, concentrated alkali is required to restrain the decomposition of formic acid and H_2_O_2_ is added to enhance the oxidation capacity. POMs and vanadium salts are two commonly used types of catalysts for oxidative cleavage of carbohydrate C-C bonds. The C-C bond scission pathways under different conditions are shown in Scheme 2. Additionally, 1-Hexanol is added as an *in situ* extracting agent to protect produced formic acid from over-oxidation, which will be discussed in detail.

**Table 1:** Researches on the production of formic acid (FA) from cellulosic biomass

Entry	Substrates	Catalyst/Base	Oxidant	*T*(K)	Time	Solvent	FA Yield	Reference
1	Glucose	LiOH	H_2_O_2_	308	8 h	Water	91.3%^a^	Wang et al.,^2^
2	Glucose	NaOH	H_2_O_2_	523	1 min	Water	75%^a^	Jin et al.,^16^
3	Glucose	HPA-5	/	433	1 min	Water	95.0%^a^	He et al.,^17^
4	Cellulose	HPA-3	O_2_	363	24 h	Water	9%^b^	Albert et al.,^18^
5	Cellulose	HPA-3 + *p*-TSA	O_2_	363	24 h	Water	23%^b^	Albert et al.,^18^
6	Glucose	[MIMPS]_4_PMo_11_VO_40_	O_2_	453	1 h	Water	54.7%^a^	Xu et al.,^19^
7	Glucose	HPA-5	O_2_	363	6 h	Water + 1-Hexanol	85%^a^	Reichert et al.,^15^
8	Glucose	NaVO_3_	O_2_	443	1 min	Water	54.1%^a^	Wei et al.,^20^
9	Glucose	HPA-5	O_2_	363	24 h	Water + methanol	72%^a^	Maerten et al.,^21^
10	Glucose	VOSO_4_	O_2_	413	3 h	Water	75%^a^	Tang et al.,^22^
11	Cellulose	NaVO_3_	O_2_	433	2 h	Water	64.9%^a^	Wang et al.,^23^

a yield based on the carbons.

b yield based on the weight.

#### Direct oxidation under alkaline hydrothermal conditions

From the green chemistry view, hydrothermal alkaline conversion of biomass is a promising strategy to produce formic acid, under which the high ion product (K_w_) and the low dielectric constant make the transformation of biomass proceed smoothly.^24^ Our lab found that alkali hydrothermal oxidation reaction is the potential way to produce formic acid by using glucose as a model compound of carbohydrate,^16^ the maximum formic acid yield (75%) can be achieved within 1 min at 523 K (Table 1, Entry 2). As shown in Scheme 2A, the aldehyde group of the glucose was easily oxidized to produce aldonic acid. Then, the C-C bond was broken to form lower molecular compounds by two possibilities:^11^ (1) the α-scission form between C1 and C2; (2) the β-scission form between C2-C3 or C4-C5. The lower aldonic acid then repeated the process until it was completely broken down into formic acid. The C-C bond breaking between C1 and C2 may be the main form of scission in the formation of formic acid.

H_2_O_2_ was an effective oxidant which can enhance formic acid selectivity.^2^ Excessive H_2_O_2_ can promote the oxidative cleavage of glucose molecules and prevent the dehydration process, leading the increase of formic acid yield.^16^ However, excessive strong oxidants would also lead to severe the decomposition of formic acid,^16^ especially under relatively high temperature. The alkali can slow down the decomposition process by forming formate, in which LiOH, NaOH, and KOH had the similar effect, while Ca(OH)_2_ performed poorly due to its low solubility.^25^

Recently, Chen et al. furtherly controlled the reactions under milder conditions, and the highest formic acid yield reached to 91.3% at room temperature (Table 1, Entry 1).^2^ The interaction between the alkali and hydrogen peroxide may be the decisive factor for a high formic acid yield. It is possible that this oxidation mainly followed a free radical mechanism rather than O_2_ gas-assisted oxidation form. As for the alkali, it also might accelerate the generation of O_2_^.^ and ^.^OH radicals from hydrogen peroxide. In all, this modified low-temperature hydrothermal conversion provides an effective and low-cost approach for the production of formic acid.

#### Catalytic oxidation by polyoxometallates catalysts

Eco-friendly Polyoxometallates (POMs), containing strong BrØnsted acidities, are widely used as catalysts for the oxidation of biomass into formic acid.^15^ Among them, keggin-type POMs (H_3+n_PV_n_Mo_12−n_O_40_ (HPA-n, n = 0∼5)) have been used as effective catalysts for the oxidation of biomass.^26^ The catalytic oxidative reactions over HPA-n can be explained by the Mars−van Krevelen theory.^26^ The anionic metal−oxygen clusters on the POMs have strong homogeneous-phase outer-sphere electron transfer effects.^27^ As shown in Scheme 2B, the C−C bond of the glucose is broken by oxygen atoms from HPA-n to produce some intermediates, mainly including glycolaldehyde, glyoxylaldehyde, and glyceraldehyde. Subsequently, the reduced HPA-n would be re-oxidized to the original state by the added molecular oxygen, so the HPA-n can be considered as an efficient oxygen carrier to support glucose oxidation. Wu et al. analyzed the state of vanadium species in HPA-5 during the looping oxidation of carbohydrates to formic acid by ^51^V NMR and EPR spectroscopies.^17^ The significant signals of vanadium species changed from V^V^ to V^IV^ after the glucose oxidation and from V^IV^ to V^V^ after HPA-5 re-oxidation, confirming the reduction and regeneration of HPA-5 catalyst during the looping conversion. A formic acid yield of 95% was achieved from glucose showing the excellent catalytic performance of HPA-5 (Table 1, Entry 3).

Cellulose needs to be hydrolyzed into glucose to overcome the solubility problem for a higher formic acid yield. Further, a lower pH may be beneficial for the oxidation of biomass substrate due to the formation of VO^2+^ species and protonated HPA-n, However, too much acidity could also accelerate the decomposition of formic acid. Therefore, it is exactly important to choose the proper acid to cooperate with HPA-n. *p*-TSA can increase the formic acid yield from 9% to 23% (Table 1, Entry 4, Entry 5).^18^ The Heteropolyanion-based ionic liquids (ILs) with –SO_3_H functionalized cations and PMo_11_VO_40_^4−^ anions improved the formic acid yield to 54.7% at 180°C and 1.0 MPa O_2_ (Table 1, Entry 6).^19^ Overall, the ILs may serve as bifunctional catalysts while the cations can promote the hydrolysis of cellulose into glucose and anions can accelerate the oxidation of glucose into formic acid, which provides a new strategy for the conversion of biomass.

Carbon dioxide is often produced as the main by-product from the over-oxidation of formic acid or a competing reaction path.^15^^,^^18^ In order to solve this challenge, Albert et al. developed an *in situ* extraction strategy to protect the produced formic acid.^15^ They applied HPA-5 as the catalyst, oxygen as the oxidant, water as the solvent, and a long-chain primary alcohol as an *in situ* extracting agent. The produced formic acid and HPA-5 were separated in the water-organic biphasic system through the extraction process to prevent the over-oxidation. As a result, the formic acid yield from glucose reached to 85% in 6 h at 363 K and under 20 bar oxygen pressure (Table 1, Entry 7). This extraction performs a simple and efficient strategy to avoid the over-oxidation of formic acid.

#### Catalytic oxidation by vanadium salt catalysts

From the catalyst design strategy, vanadium salts, typically as VOSO_4_ are also been found as efficient catalysts for the production of formic acid from carbohydrates.^22^ The yield of formic acid reached 70%–75% from glucose at 413 K in 1 h with 2 MPa of O_2_ by adding methanol or ethanol as an *in situ* extracting agent (Table 1, Entry 10). Formic acid was produced by the isomerization of glucose to fructose and subsequent oxidative cleavage of the C_3_ intermediates, as shown in Scheme 2C. It was found that CO_2_ was still the main by-product from the decarboxylation of the carboxylic acid intermediates.^28^ The *in situ* extracting agent plays a key role to improve the formic acid yield.

Additionally, NaVO_3_ was also reported to be used, but with the cooperation of H_2_SO_4_ aqueous solution in the oxygen atmosphere.^23^^,^^29^ 64.9% yield of formic acid was obtained at 433 K in 2 h with 3 MPa of O_2_ and 0.7 wt % H_2_SO_4_ by using NaVO_3_ as the catalyst (Table 1, Entry 11).^23^ The conversion process contained initial and deep hydrolysis,^29^ which were accelerated in the presence of H_2_SO_4_. The oxidations were mainly promoted by NaVO_3_ to produced formic acid from monosaccharide, which also formed acetic acid from levulinic acid. Moreover, the increase of H_2_SO_4_ concentration was conductive to the deep hydrolysis but adverse to the catalytic oxidation.^30^ It is therefore important to regulate the amount of acid and oxygen to improve the selectivity of the reaction by enhancing the catalytic oxidation and decreasing the deep hydrolysis.

Overall, how to keep the balance between oxidation and over-oxidation is the key for formic acid production. Hydrothermal reaction with the alkali catalysts may be the most selective way for producing formic acid, but high concentration of alkali will corrode the reaction vessels. The acidic POMs catalysts and Vanadium salt catalysts are also viable choices, which can accelerate subsequent oxidative cleavage but tend to over-oxidation. How to enhance the reaction selectivity is still challenging. It seems that water-organic biphasic simultaneous extraction system provides the approaching option by separating formic acid from the water phase to avoid the over-oxidation.

### Oxidation of cellulosic biomass toward acetic acid

Acetic acid is the second simplest carboxylic acid which can be applied as the acidity regulator, solvent, and raw material, whose annual global consumption is about 6.5 million tones.^1^ Acetic acid can also be produced from oxidative cleavage of cellulosic biomass. As early in 2002, Koichi et al. used hydrothermal oxidation to convert cellulosic biomass production for acetic acid, 10 and 29 mg acetic acid were directly obtained from 1 g glucose in the absence and presence of H_2_O_2_ respectively.^31^ Due to the strong oxidation of OH radicals under the hydrothermal condition, the yield of acetic acid was very low by the one-step reaction. Generally, compared with the production of formic acid, it is more difficult to regulate the selective oxidation of biomass toward acetic acid in the one-pot strategy.

**Scheme 3:**
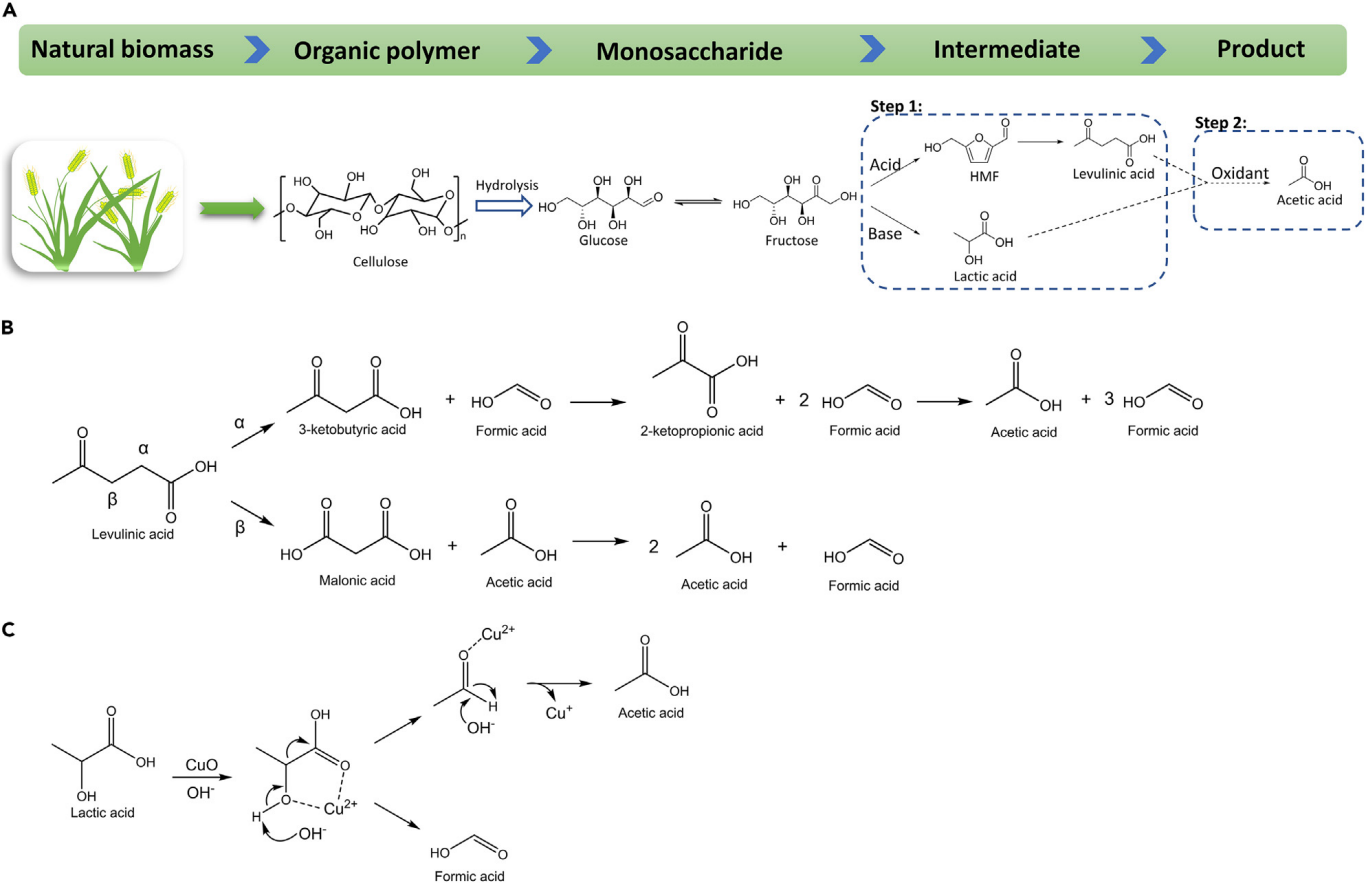
Cellulosic biomass valorization to acetic acid (A) Two-step strategy of converting cellulosic biomass into acetic acid.^32^ (B) Proposed oxidation mechanism of levulinic acid in acidic two-step conversion.^33^ (C) Proposed oxidation mechanism of lactic acid in alkaline two-step conversion with CuO as the oxidant.^34^

In order to enhance the acetic acid yield, one idea is to produce more transition products conductive to acetic acid formation before the oxidation process. Therefore, the two-step strategy is carried out to convert cellulosic biomass into acetic acid.^32^ In the first step, cellulose-derived monosaccharide is degraded into some short-chain intermediates in the absence of oxidant, and these intermediates are oxidized into acetic acid in the second step (Scheme 3A). The intermediates are variable with the addition of an acid or base. HMF and levulinic acid intermediates are formed under acidic conditions, while lactic acid intermediate is formed under alkaline conditions. By conducting the non-oxidative breaking of carbohydrates with acids or bases and oxidative cleavage of intermediates separately, two-step conversion is more selective and efficient than the one-pot reaction. Both acidic and alkaline two-step methods are introduced and discussed in the following sections.

#### Acidic two-step strategy

In the acidic two-step process, the –OH groups in monosaccharide would be protonated by Brønsted acids, leading to the formation of HMF via cleavage of corresponding C-O bonds, and then the C1-C2 bond in HMF is cleaved via a decarboxylation to produce levulinic acid intermediate.^35^ Acetic acid can be obtained from levulinic acid via oxidative cleavage of the C-C bond, and the oxidation mechanism of levulinic acid is shown in Scheme 3B. Considering the influence of highly active α-hydrogen, the most possible attack by hydroxyl radicals may occur at the α-carbon in levulinic acid molecules. Theoretically, 1 molecule of acetic acid and 3 molecules of formic acid can be obtained from 1 molecule of levulinic acid via the α-oxidation pathway. Another possible attack is at the β-carbon leading to the formation of malonic acid as the intermediate, and 2 molecules of acetic acid can be finally produced from 1 molecule of levulinic acid theoretically. Although β-oxidation may produce more acetic acid theoretically, the experimental acetic acid yield was closer to the result of the α-oxidation pathway.^33^

Mineral acids were conventionally applied to give abundant Brønsted acidity for the production of intermediates (HMF and levulinic acid) from monosaccharides in the first step of the conversion process. Experimental results of acetic acid yield showed that HCl performed better than H_2_SO_4_ and H_3_PO_4_, probably due to the better acceleration of levulinic acid intermediate formation by HCl in the first-step reaction.^33^ Furtherly, the pH should be carefully adjusted, and the highest acetic acid yield from glucose was achieved at pH = 0.5.^33^ Higher pH may suppress the conversion of HMF to levulinic acid intermediate,^36^ while lower pH may promote the degradation of acetic acid product.^33^ In the second step, moderate oxidation is required because excess oxidant may over-oxidize acetic acid toward CO_2_.^33^^,^^37^ The yield for acetic acid of 23% and 26% were respectively achieved from glucose and fructose when HCl was added (pH = 0.5) in the first step and H_2_O_2_ was applied to the second step.^33^

#### Alkaline two-step strategy

As for the alkali two-step conversion, monosaccharides are degraded to lactic acid intermediate via a retro-aldol reaction in the first step. In the oxidation step, the -C-O bond in the lactic acid would be oxidized to -C=O forming a dicarbonyl structure, which is furtherly oxidized to produce acetic acid. In contrast, the alkaline two-step conversion may be more selective than the acidic pathway, since lactic acid was reported to be more feasible to produce acetic acid than the furan intermediates.^38^ Besides the soluble strong alkalis, environmental-friendly bentonite was also applied as a solid base catalyst.^39^ Bentonite contained strong capacity of cation exchange which can lead to abundant Brønsted base sites, accelerating the conversion of biomass into lactic acid in the first step. 27% yield of acetic acid from glucose was obtained at 275°C in 100 s.^39^

Except for the selection of acids or bases in the first step, the selection of oxidants in the second step can also affect the acetic acid selectivity. Besides H_2_O_2_ and oxygen, some metal oxides have also been applied as the oxidants in the two-step reaction. For instance, CuO has been reported as an effective oxidant to produce acetic acid and it can be reduced to Cu_2_O and Cu in the second step.^34^ According to the proposed oxidation mechanism (Scheme 3C), Cu(II) species can reduce the electron density of the hydroxyl group by coordinating with the two adjacent oxygen atoms of lactic acid molecule, making it easier for OH^−^ to launch a nucleophilic attack on the hydrogen atom of the hydroxyl group.^34^ The acetic acid yields of 26%, 22% and 23% were respectively achieved from glucose, cellulose and starch via the alkali two-step process. Notably, comparing with traditional metallurgical methods, this way also provides a promising hydrometallurgy to obtain metals under much milder conditions along with organic acids.

## Retro-aldol reaction toward lactic acid and glycolic acid

### Anaerobic retro-aldol reaction for lactic acid

Lactic acid is considered as an important feedstock in the production of poly-lactic acid polymer, pharmaceuticals and cosmetic products.^40^ Traditionally, lactic acid can be produced by the microbial fermentation of biomass, which takes a long time and space.^41^ Recently, oxygen-free hydrothermal conditions are used in the production of lactic acid to avoid its further oxidation.^42^ In a typical process, the fructose can be broken for lactic acid via a retro-aldol reaction. Alkalis are usually added since OH^−^ promotes the C-C retro-aldol reactions. In some cases, lewis acid catalysts are also welcomed.

#### Alkaline catalysts

**Scheme 4:**
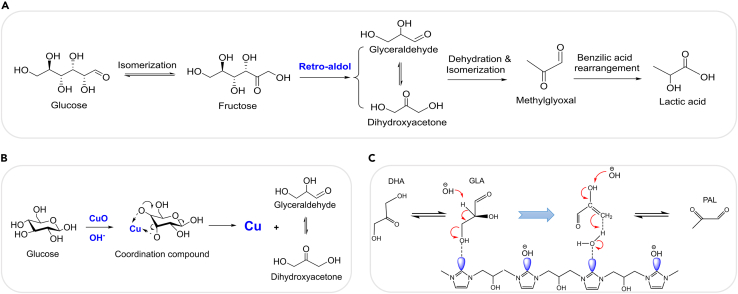
Production of lactic acid from biomass in the presence of alkali (A) Proposed pathways of producing lactic acid from glucose under alkaline conditions.^42^ (B) Retro-aldol reaction catalyzed by CuO and alkali.^43^ (C) Conversion of C3 intermediates into PAL in the presence of IMEP-based co-catalyst and alkali.^44^

In an anaerobic atmosphere, alkaline can catalyze the conversion of cellulose and its derived carbohydrates to lactate under hydrothermal conditions. Cellulosic biomass was usually applied as the substrates in recent researches, and the widely accepted pathways for this conversion of cellulose to lactic acid are shown in Scheme 4A. Firstly, glucose was formed via the hydrolysis of cellulose and then fructose was generated via the isomerization of glucose. The produced fructose was converted to glyceraldehyde and dihydroxyacetone via a retro-aldolization process, and these C3 intermediates will be converted into methylglyoxal subsequently. Finally, lactic acid was obtained from a benzilic acid rearrangement. The retro-aldolization of fructose may have the highest energy barrier among these mentioned processes.^6^ As a result, this retro-aldol process can be regarded as the rate-determining step for the production of lactic acid and alkaline catalysts can accelerate this step by lowering its energy barrier.

Alkaline catalysts such as alkali-metal hydroxides, alkali earth hydroxides and some mild-alkaline compounds were applied. Alkali-metal hydroxides, such as LiOH, NaOH, and KOH, have high efficiencies under hydrothermal conditions. Jalel et al. produced lactic acid from bread residues over alkaline catalysts and the lactate yields were 36.04%, 36.97%, 32.54% in the presence of NaOH, KOH, LiOH respectively.^45^ Besides the alkali metal hydroxides, alkali-earth metal hydroxides, such as Ca(OH)_2_, Mg(OH)_2_, Ba(OH)_2_, and Sr(OH)_2_, also have been used.^42^^,^^46^ 15%, 49%, 40%, 53% yields of lactic acid can be obtained with NaOH, Ca(OH)_2_, Sr(OH)_2_, and Ba(OH)_2_ under the same conditions.^46^ In fact, divalent cations of the alkali-earth metal hydroxides will form a transition complex, which can be beneficial to the production of lactic acid. Ba(OH)_2_ was superior, which can be applied to the transformation of glucose with a higher lactate yield even at room temperature.^42^ Additionally, to avoid their corrosion to the reaction vessels, some mild-alkaline catalysts such as Na_2_SiO_3_ was introduced to produce lactic acid from biomass, and 30% lactic acid yield was obtained from glucose at 573 K for only 60 s.^47^

An upgrading method for lactic acid is through alkalis with co-catalysts. Different from the single alkaline catalyst above, the catalytic system of alkalis with co-catalysts containing Ni, Cu and polymer presents high efficiency for the transformation of biomass. A lactate yield of 59% from glucose was obtained at 461 K for only 9 s in the presence of NaOH and CuO.^43^ As shown in Scheme 4B, the solubility of CuO was increased in the presence of the strong base such as NaOH, resulting in the formation of cupric hydroxo complex, which was from the reaction of some dissociated Cu(II) ions with the hydroxyl oxygen atoms of glucose. Cu(II) can be reduced to Cu or Cu_2_O while the glucose in the coordination compound was oxidized concurrently to the glyceraldehyde, which was a key intermediate to form lactic acid subsequently. In general, the main catalytic performance of CuO may be reflected in promoting the retro-aldolization of fructose to form glyceraldehyde or dihydroxyacetone, and the alkali can catalyze the subsequent conversion of these C3 intermediates into lactate.

The copper oxide species loaded on MgO is a useful surfactant-mediated hydrothermal strategy.^48^ 70% yield of lactate from glucose can be obtained over this Cu-CTAB/MgO catalyst in the presence of 2.5 mM NaOH at 393 K in 1 h. The catalytic capacity of Cu-based co-catalysts was improved by using proper surfactants to control the desired Cu species, which is useful for reference in the design of other metal-based catalysts.

In addition to the copper oxides, other transition metal-based species have also been introduced to the production of lactate from biomass in the presence of alkalis, such as Ni-based and Zn-based types.^49^^,^^50^ The highest yield of 58.8% for lactate was obtained at 260°C for 2 h with 1 M NaOH and 0.052 g NiO nanoplates.^50^ Besides the metal oxides species, elemental form of the metals were also used to accelerate the conversion of biomass to lactate, the highest lactate yield of 42% was achieved at 300°C for 5 min with 0.02 g of Zn, 0.03 g of Ni, 0.07 g of activated carbon and 2.5 mol/L NaOH.^49^ In general, adding transition metal-based species as the co-catalysts can not only improve the lactate yield but also decrease the amount of alkali.

It is noteworthy that polymers were also applied to the alkaline transformation of biomass into lactate as co-catalysts. IMEP-based (polymerizate of imidazole and epichlorohydrin) species, were applied as co-catalysts to the alkaline conversion of biomass into lactate.^44^ The IMEP-based co-catalysts mainly promoted the dehydration of glyceraldehyde (GLA) into pyruvaldehyde (PAL) (Scheme 4C). The cationic species in the IMEP-based compound had effective positive charge density, which strongly affected the adsorption of other negatively charged species, such as hydroxyl ions and the electronegative oxygen of GLA. The coordination with the electronegative oxygen on the GLA can facilitate the dehydration process to form lactate. The highest lactate yield of 63% was obtained from glucose over [IMEP]Cl at 100°C in 50 mM NaOH solution. Compared with the alkaline conversion process, IMEP-based co-catalysts provided a meaningful and green way to achieve high lactate yield with lower reaction temperature and lower alkali concentration.

#### Lewis acid catalysts

As an alternative, some Lewis acids were used as the catalysts in the production of lactic acid form the biomass. Generally, late transition metal salts, metal-modified zeolites, lanthanide-based and transition metal-based species were mostly introduced as the Lewis catalysts in the recent researches.^6^

**Scheme 5:**
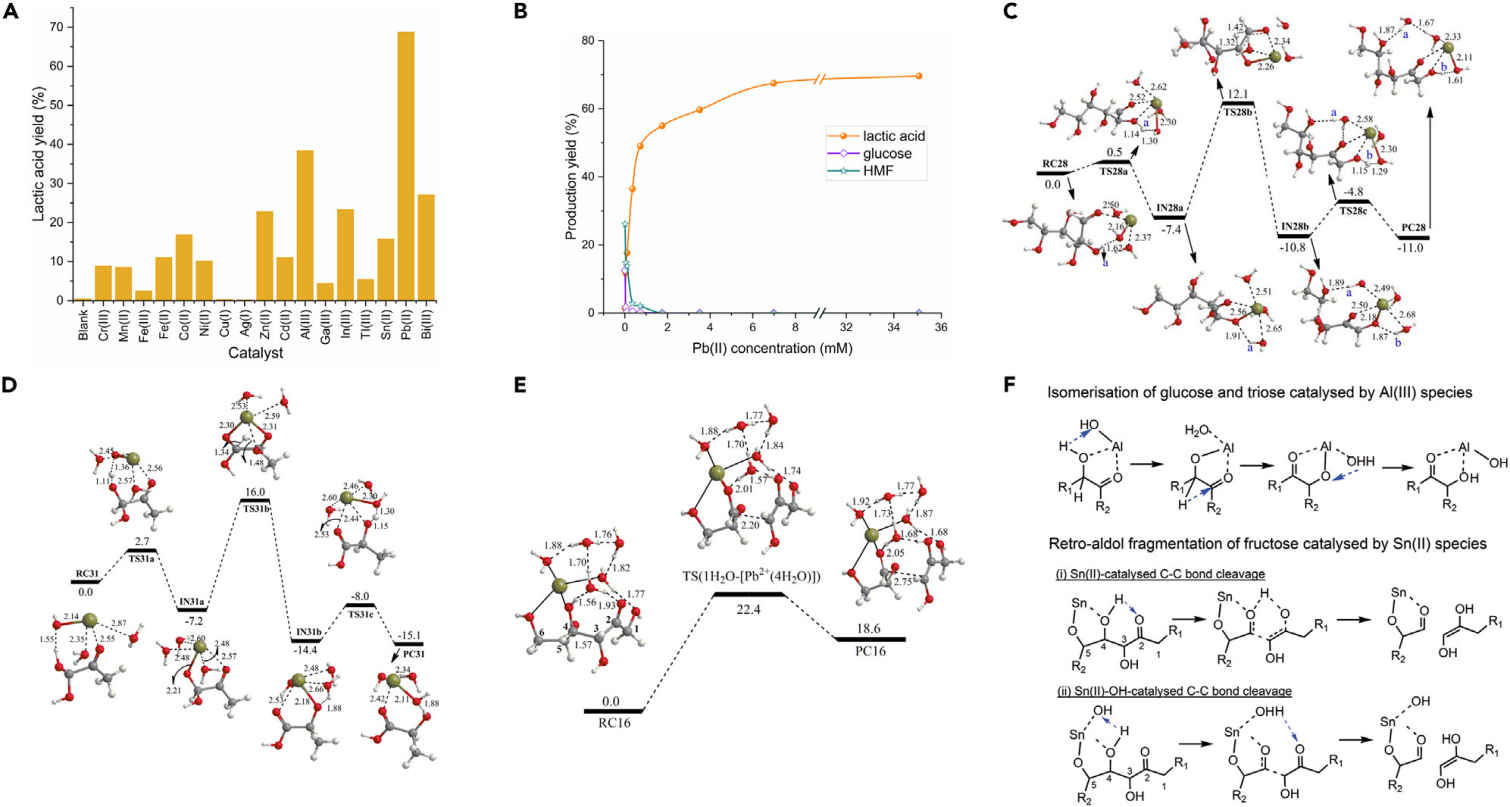
Conversion of cellulosic biomass to lactic acid catalyzed by metal salts (A) Catalytic performance of different metal cations in the lactic acid production from cellulose. Adapted with permission from Wang et al.^6^ (B) The effect of Pb(II) concentration on product yields in the cellulose conversion. Adapted with permission from Wang et al.^6^ (C) Theoretical calculation results of relative Gibbs energy profiles (in kcal mol^−1^) and optimized structures (in Å) of species for glucose isomerization using the Pb(II)-OH model. Reproduced with permission from Wang et al.^6^ Copyright 2013 Springer Nature. (D) Theoretical calculation results of relative Gibbs energy profiles (in kcal mol^−1^) and optimized structures (in Å) of species for triose isomerization using the Pb(II)-OH model. Reproduced with permission from Wang et al.^6^ Copyright 2013 Springer Nature. (E) Theoretical calculation results of relative Gibbs energy profiles (in kcal mol^−1^) and optimized structures (in Å) of species for 1H_2_O-mediated C-C bond cleavage with explicit 5H_2_O molecules catalyzed by Pb(II). Reproduced with permission from Wang et al.^6^ Copyright 2013 Springer Nature. (F) Proposed mechanism of isomerization reaction catalyzed by Al(III) species, and retro-aldol fragmentation catalyzed by Sn(II) species. Reproduced with permission from Wang et al.^40^ Copyright 2018 The Royal Society of Chemistry.

A simple and efficient strategy to produce lactic acid directly from cellulose and even cellulosic raw biomass was found by using dilute metal salts.^6^ Wang et al. discovered that Pb(II) performed better than other tested simple metal cations (Scheme 5A). The lactic acid yield increased steeply with the Pb(II) concentration while the HMF yield showed contrary tendency (Scheme 5B), suggesting that Pb(II) may hinder the competing pathway toward HMF. Through a series of theoretical calculations (e.g., Schemes 5C and 5D), the results indicated Pb(II)-OH was the active site of the Pb(II) salts, which accelerated the isomerization process including the isomerization of glucose into fructose and DHA into lactic acid via 1,2-hydride shift path. Besides, Pb(II) can catalyze the retro-aldol process by significantly lowering the activation Gibbs energy from 32.8 to 22.4 kcal/mol (Scheme 5E). Considering the toxicity of Pb(II), less toxic Al(III) and Sn(II) were introduced as a dual cation system subsequently.^40^ Al(III) mainly promoted the isomerization of glucose and the C3 intermediates, while Sn(II) took effect in the retro-aldol condensation of fructose (Scheme 5F). 81% and 66% yield lactic acid were obtained from glucose and ball-milled cellulose respectively at 463 K for 4 h.

As environment-friendly catalysts, Lanthanide-based lewis acid, in particular lanthanide metal salts and lanthanide triflates, were found efficient in the conversion of cellulose into lactic acid.^51^^,^^52^ High lactic acid yields of 91.1% and 89.6% from cellulose were achieved at 513 K for 30 min by applying ErCl_3_ and Er(OTf)_3_ as the catalysts to the reaction respectively.^51^^,^^52^ The Er-based Lewis catalysts mainly accelerated the cleavage of the protonated C-O bonds by forming coordination between hydroxyl groups and hydrated erbium ions.

Due to their recoverability, heterogeneous metal-modified zeolite catalysts have been introduced into the conversion of biomass into lactic acid.^12^^,^^53^ MWW zeolite was selected due to its pore topology. However, the strong Brønsted acidity of the MWW zeolite can decrease the selectivity toward lactic acid due to the competitive acetalization of pyruvic aldehyde. To overcome the problem, Sn was incorporated into the lattice of MWW zeolite to improve the selectivity toward lactic acid by increasing the Lewis acidity and decreasing the Brønsted acidity. The lactic acid yield of 96% from DHA was obtained at 383 K for 6 h.^53^ The additive of the Fe(III) also greatly enhanced the Lewis acidity which was suitable for the retro-aldol fragmentation reaction of ketones and the isomerization reaction of aldoses. Generally, environmental-friendly and recyclable Fe-Sn-bimetal modified Beta zeolites propose a promising strategy to convert biomass into lactic acid.^12^

Some transition metal-based species, such as Ni-based, Zn-based types, Zr-based and Nb-based types, have also been introduced as the co-catalysts with strong alkali in the above sections.^54^^,^^55^ ZrO_2_ may mainly promote the retro-aldol condensation of fructose. The C3-C4 bond of the fructose was easier to break up due to the combination between ZrO_2_ and fructose. 21.2% lactic acid yield was obtained from cellulose at 473 K for 6 h.^54^ Nb-based nanoscopic fluorides, such as Nb@AlF_3_, Nb@MgF_2_ and Nb@CaF_2_ have been found effective in the catalytic transformation of cellulose into lactic acid, which mainly promoted the isomerization of glucose and the retro-aldol condensation of fructose.^41^^,^^56^

In all, a series of efficient and cost-effective methods were developed to produce lactic acid. The alkaline catalysts can also promote the rearrangement of methylglyoxal into lactate while the Lewis acid catalysts are also suitable for the isomerization process. Since excess strong alkaline species are corrosive to the reaction vessels and environment, it seems the Lewis acid types may be more environment-friendly. Lewis acid species can change the reaction rote from the production of HMF to the production of lactic acid by lowering the activation Gibbs energy of the retro-aldol fragmentation. From the green-chemistry point of view, Lewis acid catalysts may be more suitable for the industrial production of lactic acid.

### Aerobic retro-aldol reaction for glycolic acid

Glycolic acid is a useful organic acid substrate applied to the polymer synthesis industry, especially the synthesis of polyglycolic acid fiber (PGA) for biomedical applications.^57^ The conventional manufacture of glycolic acid is via the carbonylation of formaldehyde under quite harsh conditions.^58^ In contrast, it is more attracting to obtain glycolic acid from biomass valorization. In general, the substrates can be divided into two types, one is carbohydrate biomass and the other is short-chain biomass derived platform molecules.

#### Non-oxidative cleavage of carbohydrates to glycolic acid

**Scheme 6:**
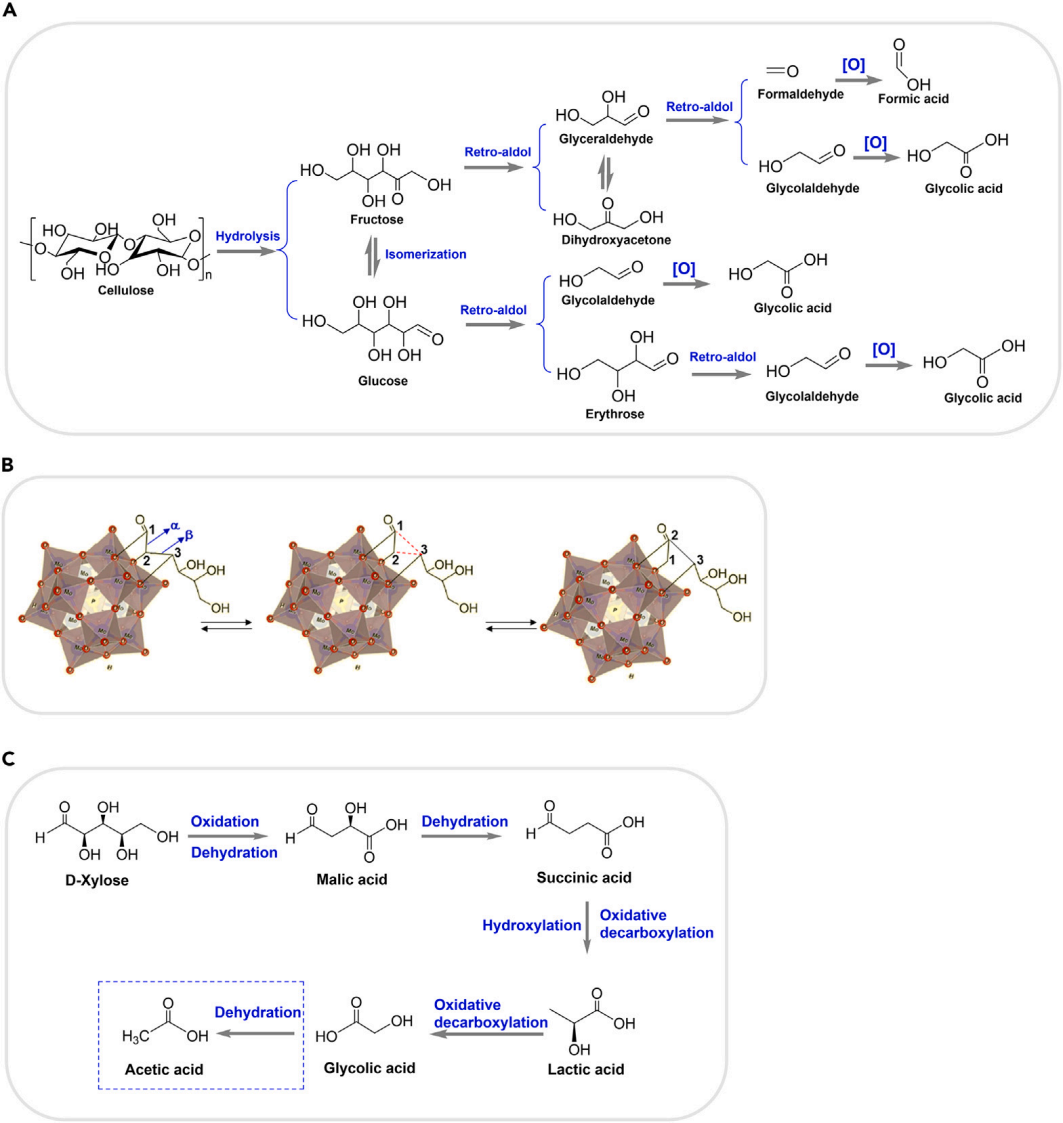
Selective oxidation of carbohydrates to glycolic acid (A) Transformation of cellulose into glycolic acid.^59^ (B) Proposed reaction pathways of epimerization and [2 + 4] retro-aldol of glucose catalyzed by phosphomolybdate ions. Reproduced with permission from Guan et al.^60^ Copyright 2018 Elsevier. (C) Production of glycolic acid from xylose over ZVO catalyst.^61^

Different from the production of lactic acid from biomass under anaerobic conditions, glycolic acid is produced under aerobic conditions. The conversion of cellulose to glycolic acid mainly involves non-oxidative cleavage of C-C bonds via retro-aldol reaction. As shown in Scheme 6A, cellulose was hydrolyzed and undergo isomerization to form fructose. By retro-aldol reaction, corresponding short-chain aldehydes were formed and glycolic acid can be produced via oxidation afterward. Formic acid can be formed as a by-product in the 3 + 3 retro-aldol pathway. In general, the retro-aldol condensations of hexoses and short-chain intermediates is the decisive steps.

Quite concentrated alkali in the atmosphere was facilitated for the conversion of polysaccharides (cellulose and starch) into glycolic acid. Early in 1988, 42 wt % yield of glycolic acid was achieved from dry cellulose at 240°C for 17 min with excessive strong alkali (16 n NaOH) by Krochta’s group.^62^ In 2012, Han et al. introduced HPA catalysts (vanadium-based) to the transformation of cellulose into glycolic acid in the presence of oxygen.^59^ Additionally, up to 49.3% and 46.5% glycolic acid yields were achieved from cellulose over H_3_PMo_12_O_40_ (HPM) and H_4_SiMo_12_O_40_ (HSM) at 453 K for 1 h. Furtherly, Guan et al. found that the HPM mainly promoted the 2 + 4 retro-aldol condensation of glucose (Scheme 6B).^60^

Besides the glucose and fructose, xylose can also be applied as the substrate to produce glycolic acid via oxidative decarboxylation. Barakat et al. proposed a microwave-assisted strategy to selectively convert xylose to glycolic acid in the presence of H_2_O_2_ as the oxidant and Zn_3_V_2_O_8_ (ZVO) as the catalyst,^61^ as shown in Scheme 6C. Malic acid, succinic acid and lactic acid were produced as main intermediates. The large amount of acid sites on the ZVO were suitable for the dehydration processes while the vanadium species catalyzed the oxidation processes. The microware accelerated these reactions probably by dispersing the reactants more evenly.^63^ 39.8% of glycolic acid yield was obtained from xylose at only 373 K for 30 min. The help of microwave under mild conditions introduces a promising and environment-friendly strategy to produce glycolic acid.

#### Mild oxidation of biomass-derived platform molecules into glycolic acid

To gain high yield of glycolic acid, some researchers tried to produce glycolic acid from biomass-derived platform molecules, such as dihydroxyacetone, glycolaldehyde, and short-chain polyols.

**Scheme 7:**
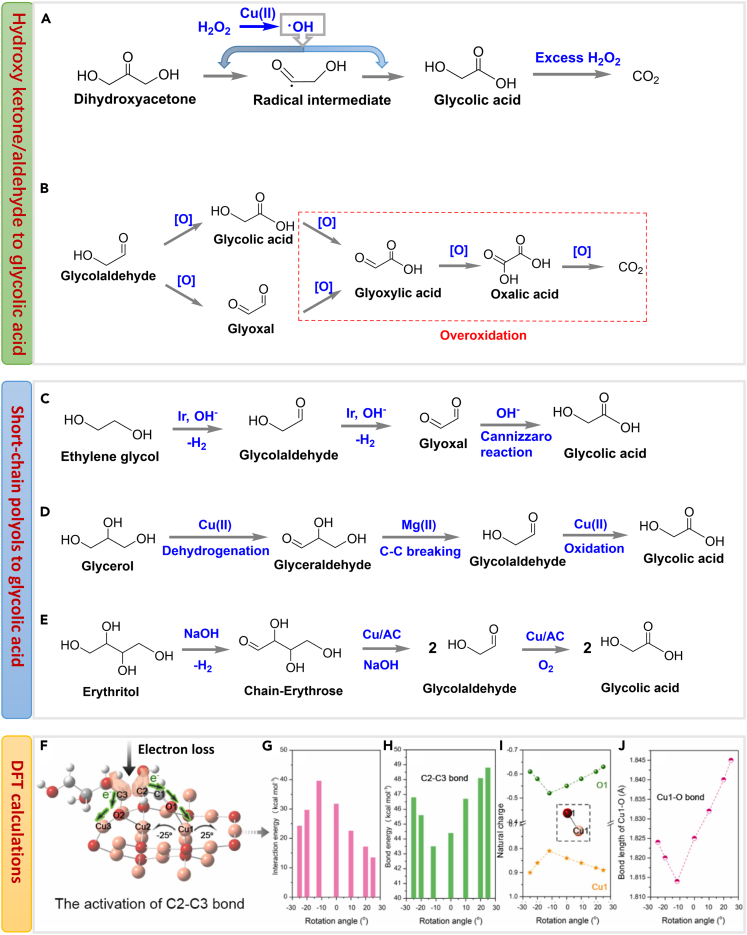
Production of glycolic acid from biomass-derived platform molecules (A) Oxidation of dihydroxyacetone into glycolic acid in the presence of H_2_O_2_ and Cu(II).^57^ (B) Catalytic oxidation of glycoladldehyde into glycolic acid and the subsequent overoxidation processes.^64^ (C) The oxidant-free conversion of ethylene glycol into glycolic acid over Ir-based catalyst in the presence of alkali.^65^ (D) Selective conversion of glycerol to glycolic acid catalyzed by Cu-Mg catalyst.^66^ (E) Selective conversion of erythritol to glycolic acid catalyzed by Cu/AC with NaOH.^67^ (F) The activation of C2-C3 bond in the transformation of polyols to glycolic acid. Reproduced with permission from Hu et al.^67^ Copyright 2022 Elsevier. (G) The effect of dangling-like Cu1-O bond deflection (−25°→25°) on the interaction energy between Cu_2_O(111) and mannitol. Reproduced with permission from Hu et al.^67^ Copyright 2022 Elsevier. (H) The bond energy of C2-C3. Reproduced with permission from Hu et al.^67^ Copyright 2022 Elsevier. (I) The natural charge analysis for O in –C1 = O of mannitol and the interacted Cu1. Reproduced with permission from Hu et al.^67^ Copyright 2022 Elsevier. (J) The bond length of Cucus-O (Å). Reproduced with permission from Hu et al.^67^ Copyright 2022 Elsevier.

Dihydroxyacetone was employed as the substrate to produce glycolic acid over Cu/Al_2_O_3_ catalyst in the presence of H_2_O_2_.^57^ Single Cu(II) species were the active sites to catalyze the formation of ^.^OH radicals from H_2_O_2_. The C-C bond in DHA was broken into radical intermediates (^.^COCH_2_OH) which instantly reacted with ^.^OH radicals to produce glycolic acid (Scheme 7A). Up to 94% glycolic acid yield was obtained from DHA at 298 K for 24 h, indicating a way to gain high yield of glycolic acid from biomass-derived DHA over economic catalyst at room temperature.

Glycolaldehyde was also selected as the substrate to produce glycolic acid by the oxidation of the aldehyde group.^68^ Mild oxidation is required to prevent the hydroxy of glycolaldehyde and produced glycolic acid from being over-oxidized (Scheme 7B). Wang et al. obtained 94.0 C-mol% yield of glycolic acid from glycolaldehyde in the presence of NaOH and Cu/AC.^67^ They proposed that the removal of H in the aldehyde group of glycolaldehyde can be facilitated due to the combination between -C=O group with Cu_2_O (1 1 1), thus enhancing the formation of glycolic acid eventually.

Additionally, biomass-derived short-chain polyols, including ethylene glycol, glycerol, and erythritol, can produce glycolic acid. Au/Al_2_O_3_^69^ and bimetallic Pt-Fe/CeO_2_^70^ efficiently catalyzed the oxidation of ethylene glycol to glycolic acid in the presence of oxygen. In addition, Tang et al. proposed an oxidant-free strategy to transform ethylene glycol into glycolic acid catalyzed by homogeneous Ir-based catalyst under alkaline conditions via a cascade dehydrogenation and Cannizzaro reaction (Scheme 7C).^65^ For glycerol and erythritol, catalytic dehydrogenative oxidation strategy is feasible in the production of α-hydroxyl acids with Cu/γ-Al_2_O_3_ and robust Cu_1_Mg_4_.^66^^,^^67^^,^^71^ Considering the completing path toward lactic acid induced by strong base,^71^ Hu et al. developed a base-free strategy to produce glycolic acid from glycerol in the presence of a multi-functional Cu-Mg catalyst.^66^ Under base-free conditions, Cu(II) species dominantly contributed to the dehydrogenation of glycerol and oxidation of glycolaldehyde, while Mg(II) species mainly promoted the cleavage of C2-C3 bond in glyceraldehyde to give glycolaldehyde (Scheme 7D).^66^ 71.8% yield of glycolic acid was obtained from glycerol over this multifunctional catalyst under optimal reaction conditions, which may be the highest value to date. As for the conversion of erythritol to glycolic acid (Scheme 7E), erthritol firstly underwent the oxidative dehydrogenation in the terminal -CHOH to -C=O to give chain-erythrose intermediate, glycolic acid can be formed through the cleavage of this C2-C3 bond via a retro-aldol reaction and oxidation of the glycolaldehyde.^67^ DFT calculation showed Cu species (Cu_2_O) may enable the precise activation of C1-H and C2-C3 bonds via directional electron transfer from polyols to Cu atom (Schemes 7F–7J).^67^ In conclusion, Cu-based catalysts provide efficient tools for the precise production of glycolic acid even under mild conditions.

## Cellulosic biomass conversion toward levulinic acid and 2,5-furandicarboxylic acid

### Dehydration of hydroxyl groups into levulinic acid

**Scheme 8:**
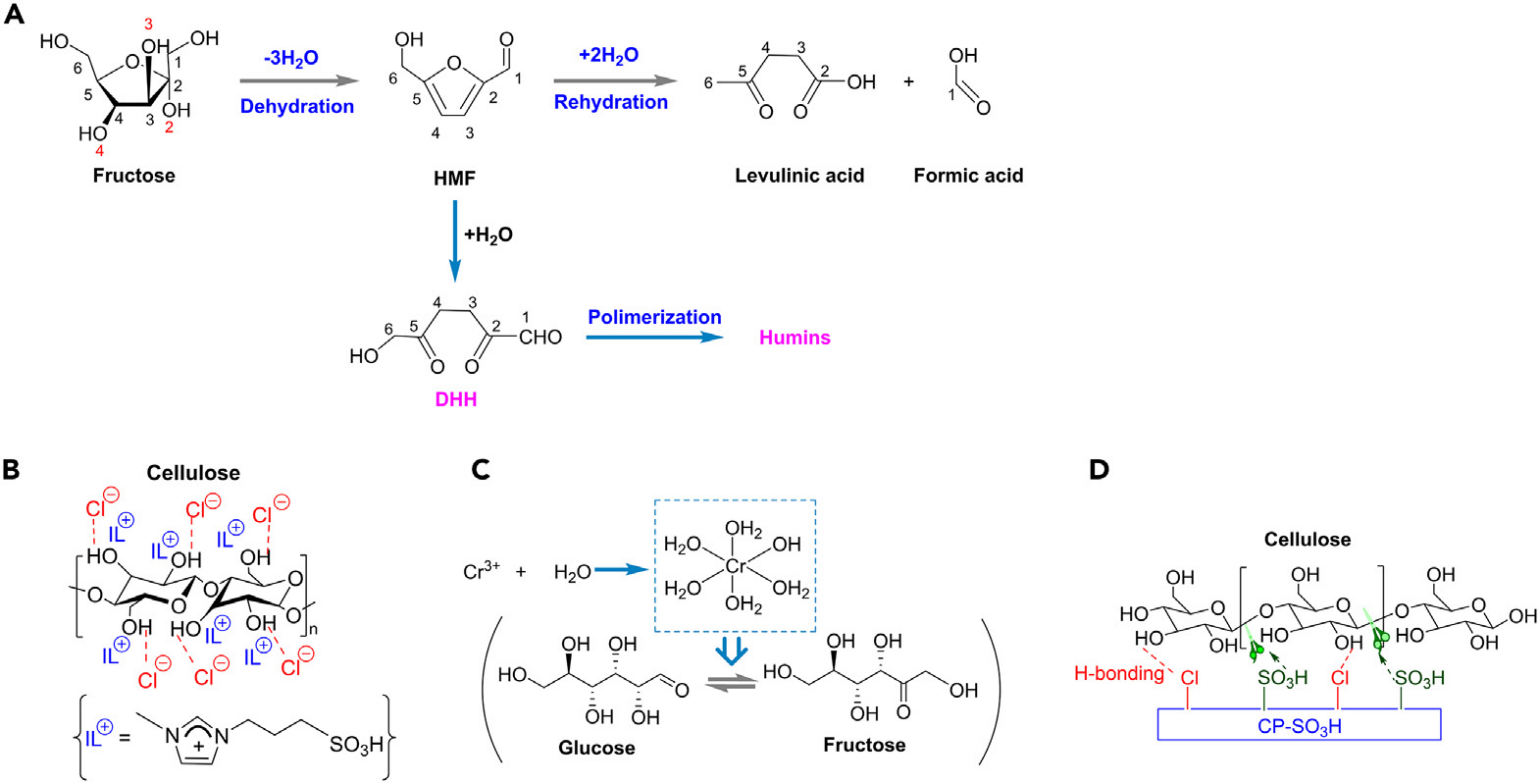
Production of levulinic acid from biomass (A) Transformation of fructose into levulinic acid under acidic condition.^73^ (B) H-bonding between ILs catalyst and cellulose biomass.^72^ (C) The isomerization of glucose to fructose catalyzed by Cr(III) species.^75^ (D) Interactions between cellulose and CP-SO_3_H catalyst.^76^

Levulinic acid, which can also be denoted as 4-oxopentanoic acid, is a valuable platform compound to produce other desired chemicals such as γ-valerolactone, alkyl levulinate biofuel additives, and polymer chemicals.^72^ In a typical chemical conversion, levulinic acid is produced from fructose by dehydrations of fructose to the HMF and rehydration of HMF to levulinic acid (Scheme 8A).^73^ Acid condition is suitable for the conversion since it can promote the protonation of glycosidic oxygen and the scission between glycosidic linkage and the nucleophilic attack of water in the rehydration process.^74^ Generally, mineral homogeneous acids (Scheme 8A), ionic liquids (Scheme 8B), metal salts (Scheme 8C), and solid acids (Scheme 8D) are the most common catalysts applied to the chemical production of levulinic acid from biomass.

#### Mineral acid catalysts

Actually, the difficulty in selective transformation for levulinic acid lies in how to promote the protonation of hydroxyl groups. As for glucose and fructose, it may be more thermodynamically favorable to obtain HMF from fructose than glucose since the unstable ring structure in fructose makes it easy to form enol by opening the chain.^77^ Therefore, the levulinic acid yield from fructose was usually higher than that from glucose, while the glucose substrate needed to be furtherly isomerized into fructose during the conversion.

Mineral acids, HCl and H_2_SO_4_ were commonly applied to the conversion. Heeres et al. proposed that the activation energy in the dehydration of fructose to HMF catalyzed by H_2_SO_4_ was slightly lower than that by HCl.^78^ Their experimental and kinetic calculation results showed a clear anion effect that the reaction rates for the fructose decomposition were higher for the SO_4_^2−^ than for the Cl^−^.^78^ Besides the hexoses, mineral acids can also catalyze the conversion of natural cellulosic biomass into levulinic acid by hydrolysis. Martins et al. proposed a strategy to produce levulinic acid from rice husks.^79^ In their research, HCl performed better than H_2_SO_4_ probably because the calcium salts in husks would react with SO_4_^2−^ to form CaSO_4_ sediment. The highest levulinic yield from rice husks was up to 59.4% in the presence of HCl at 443K for 60 min.

It should be noted that solid humins can also be formed as an undesired by-product during the production of levulinic acid from biomass under acidic conditions. When insoluble cellulosic biomass was applied as the substrate, the humins may be deposited on the residual substrate, blocking further conversion of the residual biomass. To overcome the problem, Dumesic et al. constructed a GVL-Water biphasic system.^80^ This reaction system can separate the substrate and the humins to reduce the inhibition caused by humins, which might also be applied to other biomass deconstruction processes to resolve the contradictions.

#### Ionic liquid catalysts

An attractive renewable strategy for the conversion of biomass into high valued chemicals is multi-functional ionic liquid (IL) system. H-bonding ability of anions in ILs is beneficial for the production of levulinic aicd from biomass (Scheme 8B). In general, higher yield of levulinic acid can be achieved with stronger acidity. Levulinic acid yield of 17.1 wt % was obtained from straw in the presence of [C_3_SO_3_Hmim]HSO_4_ at 180°C for 1.5 h.^72^ Higher acidic dicationic ILs, such as [C_4_(Mim)_2_] [(2HSO_4_)(H_2_SO_4_)_2_], can achieve the highest levulinic acid yield of 50.7 mol % from cellulose at 100°C for 2 h.^81^ Further research tried to improve the selectivity by Brønsted acidity of ILs ([SMIM][FeCl_4_]), in which the [FeCl_4_] promoted the isomerization of glucose into fructose and the [SMIM] was beneficial for subsequent dehydration and rehydration steps. The yield of levulinic acid from glucose reached as high as 68 wt % at 150°C for 4 h.^82^ ILs system may be a promising tool for levulinic acid.

#### Metal salt catalysts

Metal salt catalysts were alternative choices welcomed by researchers due to low corrosion. Transition metals (Cr, Co and Fe) showed a remarkable catalytic activity on the isomerization of glucose into fructose. By using CrCl_3_ together with HCl as the catalyst in aqueous media, 46% yield of levulinic acid was achieved from glucose at moderate temperatures.^75^ The hydrolyzed Cr(III) complex [Cr(H_2_O)_5_OH]^2+^ may be the most active Cr species for glucose isomerization (Scheme 8C) and HCl can promote the dehydration and rehydration reactions respectively. The mixed mineral acids (H_2_SO_4_, HCl, H_3_PO_4_) and metal salts (AlCl_3_, CuCl_2_, CrCl_3_, and FeCl_3_) catalyst system performed well, giving the activation energy of 65.4 kJ/mol for glucose dehydration into HMF and 60.6 kJ/mol for HMF rehydration into levulinic acid (H_3_PO_4_-CrCl_3_).^14^ However, chromium salts have environmental risk, which may limit their industrial use.

#### Solid acid catalysts

Solid acid catalysts were gradually attracting the attention recently, and researchers tried to design desired catalysts that can promote the protonation of hydroxyl groups while prevent further contradiction reactions. Zeolite was considered as an effective solid acid catalyst early in 1987. Over LZY zeolite, levulinic acid yield of 43.2% was achieved at 140°C for 15 h from D-fructose.^83^ The produced HMF molecules can be trapped within the LZY zeolite pore, enhancing the selectivity to levulinic acid eventually. Recently, Asmadi et al. introduced CrCl_3_ with HY zeolite for the conversion of lignocellulosic biomass into levulinic acid, 15.5 wt % of levulinic acid yield was obtained from empty fruit bunch at 145°C for 146 min.^84^

A two-step strategy was proposed to increase the levulinic acid selectivity by Ion-exchange resins such as Nafion, Amberlyst and Dowex.^13^ The whole process contained two main steps: (1) hydrothermal decomposition of cellulose to produce glucose and HMF without catalysts; (2) the further conversion of produced glucose and HMF into levulinic acid in the presence of Amberlyst 70. Multifunctional reaction system using Fe-resin (a modified Dowex 50 by cation exchange) solid acid catalyst was also found for the production of levulinic acid from microcrystalline cellulose in 5 wt % NaCl solution,^85^ in which NaCl could change crystalline cellulose into amorphous forms and the Fe-resin could promote the depolymerization into water-soluble sugars. Levulinic acid yield of 33.3 wt % was achieved from crystalline cellulose under 200°C for 5 h. This shows a promising solid acid catalyst due to its comparable activity to HCl.

Multistep processes require complex reaction system and thus are extremely high-cost. Efforts have been made to enhance the levulinic acid yield by one-step hydroprocessing. Fu et al. synthesized a novel sulfonated chloromethyl polystyrene (CP) resin by partially substituting chlorine groups of CP resin with sulfonic groups.^76^ In this one-pot production of levulinic acid from cellulose, 51.5% yield of levulinic acid was achieved at 180°C for 12 h in their research. As shown in Scheme 8D, the -Cl groups of the catalyst can absorb cellulose via strong specific interactions while the -SO_3_H acid groups of the catalyst can promote the hydrolysis of the absorbed cellulose. Additionally, Qi et al. developed a cellulase-mimetic solid acid catalyst which was also efficient and eco-friendly in the cellulose conversion to levulinic acid.^74^

Generally, the hydrolysis of cellulose into water-soluble sugars was an especially crucial step. In pioneering works, mineral acids were used to catalyze the conversion of cellulosic biomass into levulinic acid by hydrolysis. But for low corrosion and separation, metal salt catalysts and solid acid catalysts are of key interest recently. Two-step strategy was proposed to overcome the difficulty of access the crystalline cellulose with solid catalysts, however multistep processes may increase system complexity and cost. One-pot production of levulinic acid from cellulose may be more potential from industrially applied view. To design desired catalysts that can promote the sorption of cellulose via strong specific interactions is the key for levulinic acid production.

### Dehydration and subsequent oxidation for 2,5-furandicarboxylic acid

**Scheme 9:**
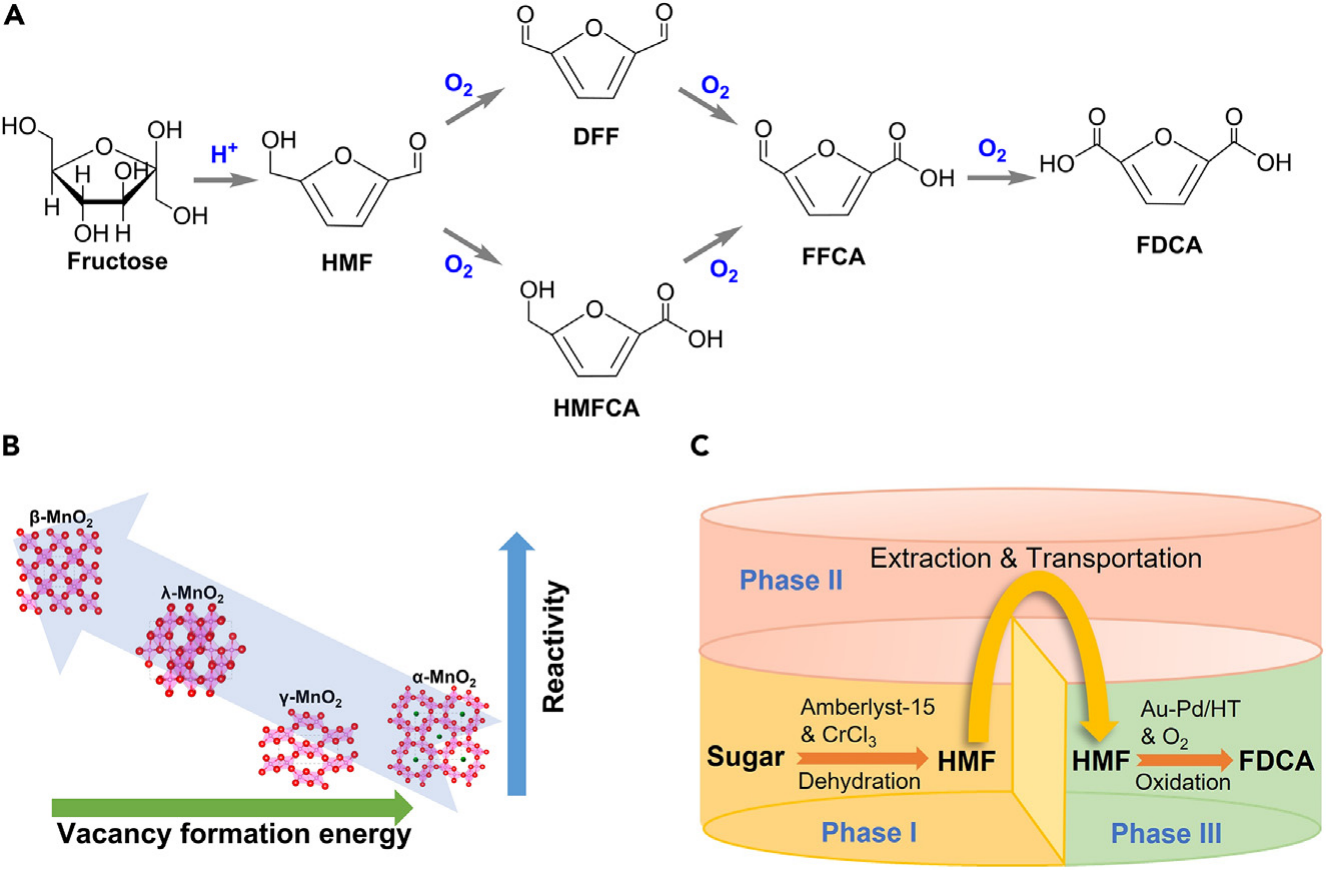
Production of FDCA from biomass (A) Proposed pathways for the production of FDCA from fructose.^3^ (B) Effect of MnO_2_ crystal structure on the conversion of HMF to FDCA. Adapted with permission from Hara et al.^86^ (C) Conversion of sugar into FDCA in triphasic reaction system.^87^

2,5-furandicarboxylic acid (FDCA) is considered as a potential environmentally friendly feedstock to replace petrochemical-derived monomers in the production of some polymers, such as polyamides, polyesters, and polyeurethanes.^3^ In a typical fructose conversion to FDCA, it mainly undergoes two steps: dehydration of fructose to HMF and oxidation of HMF to FDCA (Scheme 9A).^3^ Acidic condition is suitable for the generation of HMF from fructose, while the conversion of HMF to FDCA requires appropriate basicity, making it challenging to obtain FDCA directly from carbohydrates. As a result, how to reconcile the conflict between the reaction conditions of these two steps has become a key point for FDCA production from cellulosic biomass. Generally, the current proposed solutions can be divided into two types: (1) two-pot conversion strategy via HMF separation; (2) one-pot conversion strategy in the presence of organic solvents.

#### Two-pot catalytic conversion strategy

Since the step of HMF production and HMF conversion require quite different reaction conditions, one viable choice is to conduct these two steps in different reaction pots separately. In this strategy, HMF intermediate, which is produced from fructose dehydration in the first pot, should be separated and added to the second pot for FDCA production.

The formation of HMF from fructose requires Brønsted acid catalysts, but the produced HMF can be furtherly rehydrated into levulinic acid byproduct under acidic conditions, as discussed in the former section of levulinic acid production. Therefore, the key to the first step is to inhibit the further conversion of HMF into levulinic acid to the maximum extent. In the design of the catalyst, Brønsted acid sites are still required for fructose dehydration, but they also need to be restrained by specific designs in HMF rehydration. Rahaman et al. trapped phosphotungstic acid in the pores of MIL-101(Al)-NH_2_ to form PTA⊂MIL-101(Al)-NH_2_, a bifunctional catalyst with both Lewis and Brønsted acidity in the HMF production.^88^ The encapsulation effect of porous structure in the metal-organic framework (MOF) can prevent Brønsted acidity from leaching out into the aqueous reaction system, thus reducing the HMF rehydration. In general, trapping heteropolyacids in MOF pores provides a catalyst design strategy to regulate the behavior of Brønsted acidity. In addition to the catalysts, proper solvents can also aid the improvement of HMF selectivity. It was reported that HMF can be more stable in nonpolar organic solvents than in highly aqueous or polar solvents.^89^ Thus, the produced HMF can be extracted using an organic solvent to prevent its further transformation in the acidic aqueous phase. Water-immiscible organic solvents, such as p-xylene, diethyl ether, methylisobutylketone, or toluene, are suitable types to be applied to form such an aqueous-organic biphasic system.^90^

In the second step, HMF is firstly oxidized into DFF or HMFCA intermediate which is subsequently oxidized to FFCA, and FDCA is finally produced via further oxidation of FFCA under alkaline conditions. Strong basicity is suitable for the formation of HMFCA by promoting the Cannizzaro reaction of HMF, while the DFF formation prefers moderate basicity. Therefore, strong alkalis (e.g., NaOH, KOH) always lead to HMFCA route and moderate alkalis (e.g., Na_2_CO_3_, NaHCO_3_) contribute to DFF route. The strength of the base also affects the rate-determining reaction among HMF oxidation: HMFCA→FFCA and FFCA→FDCA is respectively the rate-determining process under strong and weak alkaline conditions.^91^^,^^92^ Besides the base, noble metal catalysts can also play a significant role in the regulation of reaction routes. Jadhav et al. applied Pd/CC to catalyze the conversion of HMF into FDCA in the presence of K_2_CO_3_, but no DFF was detected in their reaction, which excluded the possibility of DFF route.^93^ Odriozola et al. found Au/Al_2_O_3_ was efficient in the production of FDCA from HMF at high concentrations of NaOH through HMFCA route.^94^ Considering the corrosivity of strong alkalis, Schuth et al. proposed a base-free strategy to produce FDCA from HMF over Ru/ZrO_2_ catalyst via the DFF route.^95^ Overall, the reaction route can be influenced by both base and noble metal catalyst, and their regulation mechanism needs to be furtherly investigated in the future researches.

Besides the noble metals, transition metals, such as Mn and Co, were also found efficient in the production of FDCA from HMF. Hara et al. introduced different types of crystalline MnO_2_ to the oxidation of HMF into FDCA in the presence of O_2_ and NaHCO_3_.^86^ Experimental studies revealed that β-MnO_2_ had higher reactivity probably due to its lower vacancy formation energy than other tested MnO_2_ (Scheme 9B). Besides, CoO_x_ (containing metallic Co, CoO, Co_3_O_4_ species) can achieve a high FDCA yield of 95.3% from HMF at milder conditions.^96^ In addition to the monometallic types, Liu et al. synthesized a bimetal Mn-Co catalyst supported on N-doped carbon for the oxidation of HMF into FDCA.^97^ The N-doped carbon and Mn atoms can boost the activity of Co nanoparticles for the selective oxidation of HMF into FDCA via the HMFCA route. The development of efficient and cost-effective bimetal catalyst for FDCA production is promising for promoting its industrialization.

#### One-pot catalytic conversion strategy

Although two-pot conversion strategy can obtain FDCA from cellulosic biomass, there are still some disadvantages. Firstly, HMF may be difficult to be separated and transported due to its high boiling point, super hydroscopicity, and poor thermal/light stability.^98^ Secondly, two-pot conversion may be costly since more materials, energy, and equipment are required than one-pot reaction. Therefore, one-pot catalytic conversion with the help of organic solvents provides the other promising strategy for HMF production from cellulosic carbohydrates.

A triphasic system, which can complete one-pot conversion of sugars into FDCA by cleverly dividing a single reactor into different reaction regions, was successfully established by Zhang et al.^87^ Scheme 9C shows phase I-II-III, i.e., TEAB-MIBK-water or water-MIBK-water. The conversion of biomass into HMF occurred in the phase I with Amberlyst-15, then the produced HMF was extracted, purified and transferred into the phase III with the help of phase II. Besides, CrCl_3_ was added into phase I to catalyze the isomerization of glucose into fructose. Finally, the HMF in phase III would be furtherly oxidized into FDCA catalyzed by Au-Pd/HT with O_2_ bubbling under alkaline conditions. This well-designed triphasic system efficiently realized the one-pot direct conversion of sugars into FDCA with high yield under mild conditions.

Apart from the multiphase reaction system, a monophasic mixed solvent system was raised for producing FDCA directly from biomass over Pt/C catalyst in composed of g-valerolactone (GVL) and H_2_O.^3^ A high yield of FDCA was obtained with a high purity (99%) by simple crystallization. The solubility of the FDCA was improved to the maximum value in the mixed solvent at the mass ratio of GVL to water 4:1. The improvement of FDCA solubility in the mixed phase created acidic conditions to catalyze the dehydration of fructose into HMF without additional acid. Additionally, Pt/C can promote subsequent oxidation of HMF toward FDCA via the DFF route. Humins may be formed during the dehydration of fructose under acidic conditions. In general, by improving the FDCA solubility in the prepared monophasic mixed solvent, this economically viable and environmentally friendly strategy can complete one-pot direct conversion of sugars into FDCA.

## Selective oxidation of carbohydrates for sugar acids

Sugar acids, such as gluconic acid and glucaric acid are important biomass-derived chemicals for both food and pharmaceutical industries. For example, gluconic acid is used as the eco-friendly chelating agent and water-soluble cleansing agent.^99^ Glucaric acid was even listed as one of the 12 most valuable biomass derivatives by the U.S. Department of Energy.^100^ Traditionally, sugar acids are produced from biomass with the help of biological enzymes. But the concentration of the substrate in the biotechnological conversion is relatively low, limiting the amplification of this technology to the industrial scale.^99^ To meet growing demand, more attentions have been aroused about the selective oxidation of biomass to sugar acids.

**Scheme 10:**
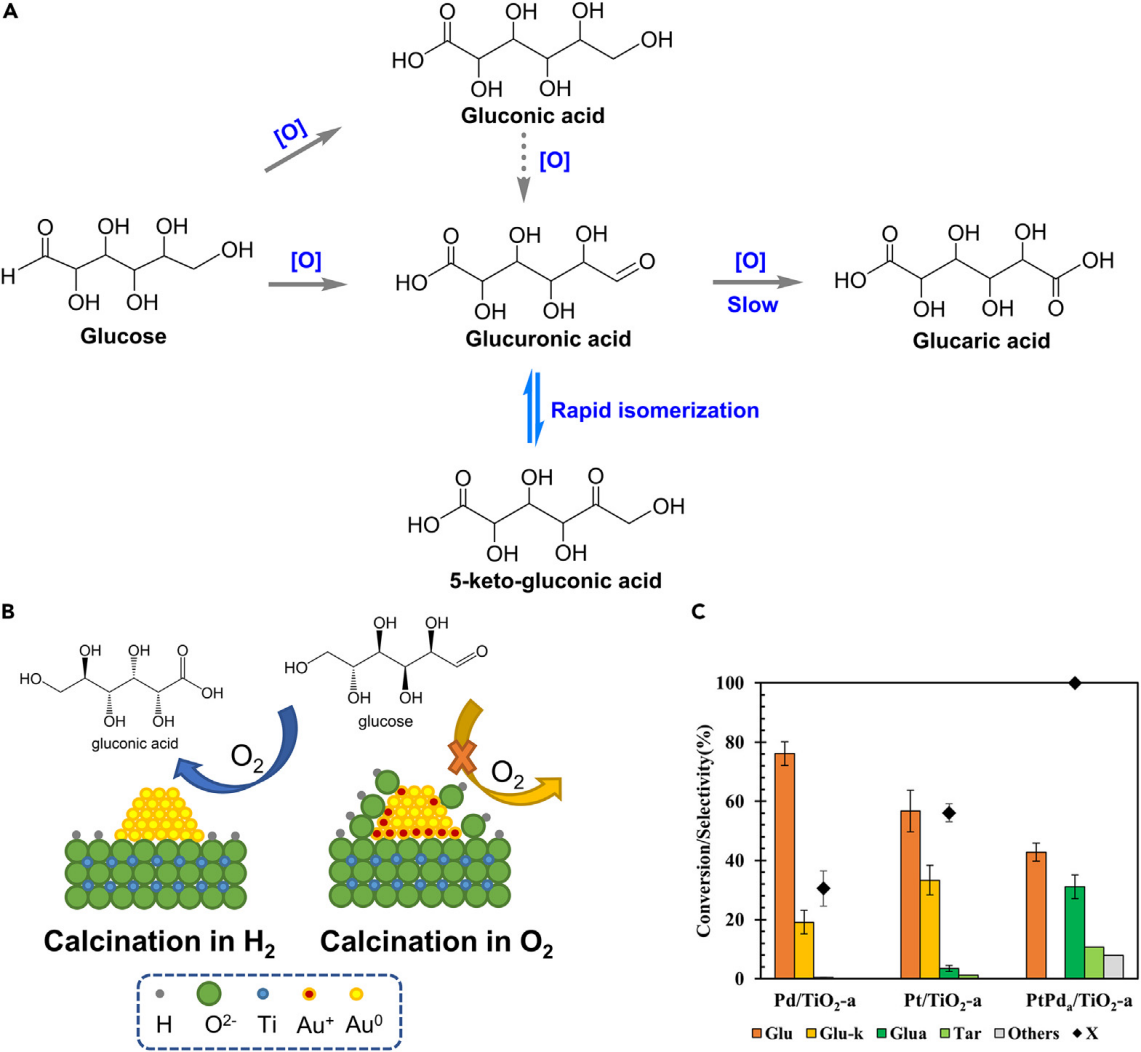
Production of sugar acids from biomass (A) Oxidation of glucose to gluconic acid and glucaric acid.^100^ (B) Oxidation of glucose to gluconic acid over Au/TiO_2_ catalyst.^101^ (C) Products selectivity in the oxidation of glucose to glucaric acid over Pd/TiO_2_-a, Pt/TiO_2_-a, and PtPda/TiO_2_-a catalysts (Glu, gluconic acid; Glu-k, 5-keto-gluconic acid; Gla, glucaric acid; Tar, tartronic acid. Others: oxalic, glyceric, lactic, glycolic, and formic acids). Reproduced with permission from Chaudhari et al.^100^ Copyright 2016 American Chemical Society.

Typical conversion of cellulose to gluconic acid and glucaric acid need cleave the glycosidic bonds of cellulose to release the glucose units via hydrolysis, then these sugar acids are produced by the selective oxidation of glucose (Scheme 10). The oxidation of –CHO for gluconic acid is more thermodynamically favourable than that of –OH to form glucaric acid, leading to the fact that the yield of gluconic acid is higher than glucaric acid. The pH and redox environment are extremely important since too much acidity may lead to undesired cleavage of C-O bonds while the strong base can enhance the cleavage of C-C bonds via retro-aldol reactions. To regulate over-oxidation and oxidative cleavage of C-C bonds, the recent developments focus more on the development of heterogeneous catalysts.

### Gluconic acid from the oxidation of aldehyde group

Gluconic acid is produced when the aldehyde group in the glucose is oxidized into carboxy group. The biggest challenge is to avoid the occurrence of other side reaction pathways toward undesired products. Alkaline condition is suitable for the oxidation reactions and can avoid the formation of HMF. However, too much basicity may also lead to other side reactions such as isomerization, retro-aldol condensation and Cannizzaro reactions. Unlike previous investigation, recent researches have been focused on the base-free oxidation of glycose to produce gluconic acid by designing proper heterogeneous catalysts, including supports.

Au has been confirmed to be more efficient in the production of gluconic acid than Pd and Pt. Simultaneously, metal oxides and carbon materials were widely used as the supports to synthesize loaded Au catalysts. Au particles treated with H_2_ annealing performed better than those calcined in air, indicating the oxidation of glucose may preferentially take place on Au^0^ species (Scheme 10B).^101^ The maximum catalytic activity (86.0%, gluconic acid yield) was obtained over 7.6 nm Au/PVA.^102^ Besides, several typical supports, such as TiO_2_, Al_2_O_3_, ZrO_2_, CeO_2_ were suitable,^101^ but higher Lewis acidity may improve the selectivity toward lactic acid rather than gluconic acid.^99^

Porous structures confinement displayed excellent performance for retarding metal and promoting interfacial reactions. Carbon nanotube, mesoporous carbon (CMK-3) and POM can be used to support Au NPs for the selective oxidation of cellobiose into gluconic acid.^103^^,^^104^ Modified by acidic functional groups, these supports can provide solid acid sites for selective cellobiose hydrolysis and furtherly modulate the electronic structures at the interface between support and Au NPs. A gluconic acid yield of 97% with >99% selectivity was obtained from cellobiose over Au/POM.^103^ High efficient supports can decrease Au loading to an acceptable amount, offering potential for the large-scale production of gluconic acid under base-free conditions.

### Glucaric acid from the oxidation of alcohol group

It is more difficult to produce glucaric acid from the oxidation of glucose than gluconic acid. As shown in Scheme 10A, gluconic acid is dehydrogenated into glucuronic acid, which can be further oxidized into glucaric acid. Besides, 5-keto-gluconic acid may be formed by rapid isomerization of the glucuronic acid. In order to improve the consecutive oxidation of gluconic acid toward glucaric acid, higher pressure of O_2_ is required. Vlachos et al. reported a relatively high glucaric acid yield (41%) from glucose over Pt/C catalyst under carefully regulated optimal reaction conditions.^105^ Moreover, acidic condition was not suitable for the further oxidation of gluconic acid and basic condition may lead to undesired C-C cleavage to various by-products with low carbons.

As a result, immense attention has been paid to develop catalysts for the selective oxidation of primary alcohols. By introducing other metals, bimetallic ones seem to be an advanced choice for the selective production of glucaric acid. For instance, bimetallic PtPd/TiO_2_ increased the yield of glucaric acid while monometallic Pt/TiO_2_ and Pd/TiO_2_ produced 5-keto-gluconic acid (Scheme 10C).^100^ In addition, some non-noble transition metals can also be introduced as the second metal with another noble metal to reduce economic cost. The Cu species can enhance the oxidation rate of C-O to C=O, decreasing the content of Pt and lowering the required oxygen pressure.^106^ As a new solution, different structures (core-shell structure, cluster-in-cluster structure) can exhibit superior capability of electronic regulation, which may be a promising tool for the selective production of glucaric acid in the future.

## Conclusions and perspectives

We summarize proposed pathways about catalytic conversion of cellulosic biomass to several typical value-added organic acids. Cellulosic biomass and its hydrolysis derivates with aliphatic poly-hydroxyl structures, may be the most suitable biomass feedstocks to produce organic acids. By controlling a series of key reactions including hydrolysis, isomerization, retro-aldol, benzilic acid rearrangement, dehydration, and rehydration, the pathways can lead toward formic acid, glycolic acid, and sugar acids under oxidation conditions, while lactic acid and levulinic acid under suitable anaerobic environment. Specifically, organic acids such as FDCA and levulinic acid derived from HMF intermediate require acid conditions, meanwhile formic acid, lactic acid, and glycolic acid which mainly come from the retro-aldol of hexoses need alkali conditions. Both acid and alkali conditions are suitable for obtaining acetic acid, but sugar acids prefer base-free production routes.

Designing efficient and selective catalysts is still essential in breaking biomass for desired products. In general, Brønsted acid catalysts are helpful to hydrolysis, dehydration, and rehydration, Lewis acid catalysts promote the reaction of isomerization and retro-aldol, and Brønsted base catalysts can accelerate the reaction of isomerization, retro-aldol, and benzilic acid rearrangement. Additionally, multifunctional catalysts are favorable for the one-pot biomass conversion. Thus, creating multi catalytic sites by nano-designing engineering is a promising strategy for the selective production of organic acids from cellulosic biomass.

Despite these tremendous achievements, there are still a lot of efforts required to go for the industrial production of organic acids from biomass. The great challenge indeed exists in scaling up the conversion from the laboratory level to the industrial level, including the stability of the industrial conversion, the economic supply chains of raw biomass feedstocks, the high purity of the products and so on. In addition, some information should be strengthened, for instance, obtaining chemicals from direct natural biomass materials (e.g., rice husk and corn straw) by simple process, deeply investigating the catalytic mechanisms at the molecular level, designing stable and efficient catalysts, assessing the environmental impacts and economic feasibility at different scales. In order to improve the economic feasibility of large-scale production, the production cost may be reduced via optimizing raw material supply chain, improving the pretreatment efficiency of raw biomass, recovering catalysts, and recycling waste heat during the process.

## References

[1] Zhang Z., Huber G.W. (2018). Catalytic oxidation of carbohydrates into organic acids and furan chemicals. Chem. Soc. Rev..

[2] Wang C., Chen X., Qi M., Wu J., Gözaydın G., Yan N., Zhong H., Jin F. (2019). Room temperature, near-quantitative conversion of glucose into formic acid. Green Chem..

[3] Motagamwala A.H., Won W., Sener C., Alonso D.M., Maravelias C.T., Dumesic J.A. (2018). Toward biomass-derived renewable plastics: Production of 2,5-furandicarboxylic acid from fructose. Sci. Adv..

[4] Zhang W.-M., Feng K.-W., Hu R.-G., Guo Y.-J., Li Y. (2023). Visible-light-induced iron redox-catalyzed selective transformation of biomass into formic acid. Chem.

[5] Wang M., Ma J., Liu H., Luo N., Zhao Z., Wang F. (2018). Sustainable Productions of Organic Acids and Their Derivatives from Biomass via Selective Oxidative Cleavage of C–C Bond. ACS Catal..

[6] Wang Y., Deng W., Wang B., Zhang Q., Wan X., Tang Z., Wang Y., Zhu C., Cao Z., Wang G., Wan H. (2013). Chemical synthesis of lactic acid from cellulose catalysed by lead(II) ions in water. Nat. Commun..

[7] Zhou X.-L., Zhang H., Shao L.-M., Lü F., He P.-J. (2021). Preparation and Application of Hierarchical Porous Carbon Materials from Waste and Biomass: A Review. Waste Biomass Valori.

[8] Song C., Zhang C., Zhang S., Lin H., Kim Y., Ramakrishnan M., Du Y., Zhang Y., Zheng H., Barceló D. (2020). Thermochemical liquefaction of agricultural and forestry wastes into biofuels and chemicals from circular economy perspectives. Sci. Total Environ..

[9] Li S., Chen G. (2020). Agricultural waste-derived superabsorbent hydrogels: Preparation, performance, and socioeconomic impacts. J. Clean. Prod..

[10] Voß D., Pickel H., Albert J. (2019). Improving the Fractionated Catalytic Oxidation of Lignocellulosic Biomass to Formic Acid and Cellulose by Using Design of Experiments. ACS Sustain. Chem. Eng..

[11] Jin F., Enomoto H. (2011). Rapid and highly selective conversion of biomass into value-added products in hydrothermal conditions: Chemistry of acid/base-catalysed and oxidation reactions. Energy Environ. Sci..

[12] Xia M., Shen Z., Gu M., Chen W., Dong W., Zhang Y. (2021). Efficient catalytic conversion of microalgae residue solid waste into lactic acid over a Fe-Sn-Beta catalyst. Sci. Total Environ..

[13] Weingarten R., Conner W.C., Huber G.W. (2012). Production of levulinic acid from cellulose by hydrothermal decomposition combined with aqueous phase dehydration with a solid acid catalyst. Energy Environ. Sci..

[14] Weiqi W., Shubin W. (2017). Experimental and kinetic study of glucose conversion to levulinic acid catalyzed by synergy of Lewis and Brønsted acids. Chem. Eng. J..

[15] Reichert J., Brunner B., Jess A., Wasserscheid P., Albert J. (2015). Biomass oxidation to formic acid in aqueous media using polyoxometalate catalysts – boosting FA selectivity by in-situ extraction. Energy Environ. Sci..

[16] Jin F., Yun J., Li G., Kishita A., Tohji K., Enomoto H. (2008). Hydrothermal conversion of carbohydrate biomass into formic acid at mild temperatures. Green Chem..

[17] He Z., Hou Y., Li H., Wei J., Ren S., Wu W. (2023). Novel chemical looping oxidation of biomass-derived carbohydrates to super-high-yield formic acid using heteropolyacids as oxygen carrier. Renew. Energy.

[18] Albert J., Wölfel R., Bösmann A., Wasserscheid P. (2012). Selective oxidation of complex, water-insoluble biomass to formic acid using additives as reaction accelerators. Energy Environ. Sci..

[19] Xu J., Zhang H., Zhao Y., Yang Z., Yu B., Xu H., Liu Z. (2014). Heteropolyanion-based ionic liquids catalysed conversion of cellulose into formic acid without any additives. Green Chem..

[20] Wei G.-H., Lu T., Liu H.-Y., Bai J.-X., Wang Q., Li G.-Y., Liang Y.-H. (2023). Exploring the continuous cleavage-oxidation mechanism of the catalytic oxidation of cellulose to formic acid: A combined experimental and theoretical study. Fuel.

[21] Maerten S., Kumpidet C., Voß D., Bukowski A., Wasserscheid P., Albert J. (2020). Glucose oxidation to formic acid and methyl formate in perfect selectivity. Green Chem..

[22] Tang Z., Deng W., Wang Y., Zhu E., Wan X., Zhang Q., Wang Y. (2014). Transformation of Cellulose and its Derived Carbohydrates into Formic and Lactic Acids Catalyzed by Vanadyl Cations. ChemSusChem.

[23] Wang W., Wang Y.P., Wu Y., Yang X., Wu Y., Liu Q., Ren S., Marsh K.N. (2014). Catalytic conversion of biomass-derived carbohydrates to formic acid using molecular oxygen. Opt. Lett..

[24] Peterson A.A., Vogel F., Lachance R.P., Fröling M., Antal Jr M.J., Tester J.W. (2008). Thermochemical biofuel production in hydrothermal media: A review of sub- and supercritical water technologies. Energy Environ. Sci..

[25] Yun J., Jin F., Kishita A., Tohji K., Enomoto H. (2010). Formic acid production from carbohydrates biomass by hydrothermal reaction. J Phys Conf Ser.

[26] Khenkin A.M., Leitus G., Neumann R. (2010). Electron Transfer−Oxygen Transfer Oxygenation of Sulfides Catalyzed by the H_5_PV_2_Mo_10_O_40_ Polyoxometalate. J. Am. Chem. Soc..

[27] Weinstock I.A. (1998). Homogeneous-Phase Electron-Transfer Reactions of Polyoxometalates. Chem. Rev..

[28] Liu J., Du Z., Yang Y., Lu T., Lu F., Xu J. (2012). Catalytic Oxidative Decarboxylation of Malic Acid into Dimethyl Malonate in Methanol with Dioxygen. ChemSusChem.

[29] Niu M., Hou Y., Ren S., Wang W., Zheng Q., Wu W. (2015). The relationship between oxidation and hydrolysis in the conversion of cellulose in NaVO_3_–H_2_SO_4_ aqueous solution with O_2_. Green Chem..

[30] Niu M., Hou Y., Ren S., Wu W., Marsh K.N. (2015). Conversion of wheat straw into formic acid in NaVO_3_–H_2_SO_4_ aqueous solution with molecular oxygen. Green Chem..

[31] Quitain A.T., Faisal M., Kang K., Daimon H., Fujie K. (2002). Low-molecular-weight carboxylic acids produced from hydrothermal treatment of organic wastes. J. Hazard Mater..

[32] Jin F., Kishita A., Moriya T., Enomoto H., Sato N. (2002). A New Process for Producing Ca/Mg Acetate Deicer with Ca/Mg Waste and Acetic Acid Produced by Wet Oxidation of Organic Waste. Chem. Lett..

[33] Fang Y., Zeng X., Yan P., Jing Z., Jin F. (2012). An Acidic Two-Step Hydrothermal Process To Enhance Acetic Acid Production from Carbohydrate Biomass. Ind. Eng. Chem. Res..

[34] Huo Z., Fang Y., Yao G., Zeng X., Ren D., Jin F. (2015). Improved two-step hydrothermal process for acetic acid production from carbohydrate biomass. J. Energy Chem..

[35] Ramli N.A.S., Amin N.A.S. (2016). Kinetic study of glucose conversion to levulinic acid over Fe/HY zeolite catalyst. Chem. Eng. J..

[36] Körner P., Jung D., Kruse A. (2019). Influence of the pH Value on the Hydrothermal Degradation of Fructose. ChemistryOpen.

[37] Lin H., Strull J., Liu Y., Karmiol Z., Plank K., Miller G., Guo Z., Yang L. (2012). High yield production of levulinic acid by catalytic partial oxidation of cellulose in aqueous media. Energy Environ. Sci..

[38] Jin F., Zhou Z., Moriya T., Kishida H., Higashijima H., Enomoto H. (2005). Controlling Hydrothermal Reaction Pathways To Improve Acetic Acid Production from Carbohydrate Biomass. Environ. Sci. Technol..

[39] Gao X., Zhong H., Yao G., Guo W., Jin F. (2016). Hydrothermal conversion of glucose into organic acids with bentonite as a solid-base catalyst. Catal. Today.

[40] Deng W., Wang P., Wang B., Wang Y., Yan L., Li Y., Zhang Q., Cao Z., Wang Y. (2018). Transformation of cellulose and related carbohydrates into lactic acid with bifunctional Al(iii)–Sn(ii) catalysts. Green Chem..

[41] Coman S.M., Verziu M., Tirsoaga A., Jurca B., Teodorescu C., Kuncser V., Parvulescu V.I., Scholz G., Kemnitz E. (2015). NbF5–AlF3 Catalysts: Design, Synthesis, and Application in Lactic Acid Synthesis from Cellulose. ACS Catal..

[42] Li L., Shen F., Smith R.L., Qi X. (2017). Quantitative chemocatalytic production of lactic acid from glucose under anaerobic conditions at room temperature. Green Chem..

[43] Wang Y., Jin F., Sasaki M., Wahyudiono Wang F., Wang F., Jing Z., Goto M. (2013). Selective conversion of glucose into lactic acid and acetic acid with copper oxide under hydrothermal conditions. AIChE J..

[44] Wang X., Song Y., Huang C., Liang F., Chen B. (2014). Lactic acid production from glucose over polymer catalysts in aqueous alkaline solution under mild conditions. Green Chem..

[45] Sánchez C., Serrano L., Llano-Ponte R., Labidi J. (2014). Bread residues conversion into lactic acid by alkaline hydrothermal treatments. Chem. Eng. J..

[46] Esposito D., Antonietti M. (2013). Chemical Conversion of Sugars to Lactic Acid by Alkaline Hydrothermal Processes. ChemSusChem.

[47] Duo J., Zhang Z., Yao G., Huo Z., Jin F. (2016). Hydrothermal conversion of glucose into lactic acid with sodium silicate as a base catalyst. Catal. Today.

[48] Choudhary H., Ebitani K. (2015). A Convenient Surfactant-Mediated Hydrothermal Approach to Control Supported Copper Oxide Species for Catalytic Upgrading of Glucose to Lactic Acid. ChemNanoMat.

[49] Zhang S., Jin F., Hu J., Huo Z. (2011). Improvement of lactic acid production from cellulose with the addition of Zn/Ni/C under alkaline hydrothermal conditions. Bioresour. Technol..

[50] Younas R., Zhang S., Zhang L., Luo G., Chen K., Cao L., Liu Y., Hao S. (2016). Lactic acid production from rice straw in alkaline hydrothermal conditions in presence of NiO nanoplates. Catal. Today.

[51] Lei X., Wang F.-F., Liu C.-L., Yang R.-Z., Dong W.-S. (2014). One-pot catalytic conversion of carbohydrate biomass to lactic acid using an ErCl_3_ catalyst. Appl. Catal. A Gen.

[52] Liu D., Kim K.H., Sun J., Simmons B.A., Singh S. (2018). Cascade Production of Lactic Acid from Universal Types of Sugars Catalyzed by Lanthanum Triflate. ChemSusChem.

[53] Guo Q., Fan F., Pidko E.A., van der Graaff W.N.P., Feng Z., Li C., Hensen E.J.M. (2013). Highly Active and Recyclable Sn-MWW Zeolite Catalyst for Sugar Conversion to Methyl Lactate and Lactic Acid. ChemSusChem.

[54] Wattanapaphawong P., Reubroycharoen P., Yamaguchi A. (2017). Conversion of cellulose into lactic acid using zirconium oxide catalysts. RSC Adv..

[55] Wattanapaphawong P., Sato O., Sato K., Mimura N., Reubroycharoen P., Yamaguchi A. (2017). Conversion of Cellulose to Lactic Acid by Using ZrO_2_–Al_2_O_3_ Catalysts. Catalysts.

[56] Verziu M., Serano M., Jurca B., Parvulescu V.I., Coman S.M., Scholz G., Kemnitz E. (2018). Catalytic features of Nb-based nanoscopic inorganic fluorides for an efficient one-pot conversion of cellulose to lactic acid. Catal. Today.

[57] Dai X., Adomeit S., Rabeah J., Kreyenschulte C., Brückner A., Wang H., Shi F. (2019). Sustainable Co-Synthesis of Glycolic Acid, Formamides and Formates from 1, 3-Dihydroxyacetone by a Cu/Al_2_O_3_ Catalyst with a Single Active Sites. Angew. Chem. Int. Ed..

[58] Samantaray P.K., Little A., Haddleton D.M., McNally T., Tan B., Sun Z., Huang W., Ji Y., Wan C. (2020). Poly(glycolic acid) (PGA): a versatile building block expanding high performance and sustainable bioplastic applications. Green Chem..

[59] Zhang J., Liu X., Sun M., Ma X., Han Y. (2012). Direct Conversion of Cellulose to Glycolic Acid with a Phosphomolybdic Acid Catalyst in a Water Medium. ACS Catal..

[60] Bayu A., Karnjanakom S., Yoshida A., Kusakabe K., Abudula A., Guan G. (2019). Polyoxomolybdates catalysed cascade conversions of cellulose to glycolic acid with molecular oxygen via selective aldohexoses pathways (an epimerization and a [2+4] Retro-aldol reaction). Catal. Today.

[61] Khallouk K., Solhy A., Idrissi N., Flaud V., Kherbeche A., Barakat A. (2020). Microwave-assisted selective oxidation of sugars to carboxylic acids derivatives in water over zinc-vanadium mixed oxide. Chem. Eng. J..

[62] Krochta J.M., Tillin S.J., Hudson J.S. (1988). Thermochemical conversion of polysaccharides in concentrated alkali to glycolic acid. Appl. Biochem. Biotechnol..

[63] Lamaa D., Messe E., Gandon V., Alami M., Hamze A. (2019). Toward a Greener Barluenga–Valdés Cross-Coupling: Microwave-Promoted C–C Bond Formation with a Pd/PEG/H_2_O Recyclable Catalytic System. Org. Lett..

[64] Liu J., Zhu X., Cao Z., Huang L., Liao Z., Xu H., Shen W. (2019). Selective Oxidation of Glyoxal to Glyoxalic Acid by Air over Mesoporous Silica Supported Pd Catalysts. Catal. Lett..

[65] Zhan Y., Hou W., Li G., Shen Y., Zhang Y., Tang Y. (2019). Oxidant-Free Transformation of Ethylene Glycol toward Glycolic Acid in Water. ACS Sustain. Chem. Eng..

[66] Xu S., Xiao Y., Zhang W., Liao S., Yang R., Li J., Hu C. (2022). Relay catalysis of copper-magnesium catalyst on efficient valorization of glycerol to glycolic acid. Chem. Eng. J..

[67] Li J., Yang R., Xu S., Zhou C., Xiao Y., Hu C., Tsang D.C. (2022). Biomass-derived polyols valorization towards glycolic acid production with high atom-economy. Appl. Catal. B.

[68] Faveere W.H., Van Praet S., Vermeeren B., Dumoleijn K.N.R., Moonen K., Taarning E., Sels B.F. (2021). Toward Replacing Ethylene Oxide in a Sustainable World: Glycolaldehyde as a Bio-Based C2 Platform Molecule. Angew. Chem. Int. Ed..

[69] Berndt H., Pitsch I., Evert S., Struve K., Pohl M.M., Radnik J., Martin A. (2003). Oxygen adsorption on Au/Al_2_O_3_ catalysts and relation to the catalytic oxidation of ethylene glycol to glycolic acid. Appl. Catal. A Gen.

[70] Shi H., Yin X., Subramaniam B., Chaudhari R.V. (2019). Liquid-Phase Oxidation of Ethylene Glycol on Pt and Pt–Fe Catalysts for the Production of Glycolic Acid: Remarkable Bimetallic Effect and Reaction Mechanism. Ind. Eng. Chem. Res..

[71] Xu S., Tian Q., Xiao Y., Zhang W., Liao S., Li J., Hu C. (2022). Regulating the competitive reaction pathway in glycerol conversion to lactic acid/glycolic acid selectively. J. Catal..

[72] Liu L., Li Z., Hou W., Shen H. (2018). Direct conversion of lignocellulose to levulinic acid catalyzed by ionic liquid. Carbohydr. Polym..

[73] Velasco Calderón J.C., Jiang S., Mushrif S.H. (2021). Understanding the Effect of Solvent Environment on the Interaction of Hydronium Ion with Biomass Derived Species: A Molecular Dynamics and Metadynamics Investigation. ChemPhysChem.

[74] Shen F., Smith R.L., Li L., Yan L., Qi X. (2017). Eco-friendly Method for Efficient Conversion of Cellulose into Levulinic Acid in Pure Water with Cellulase-Mimetic Solid Acid Catalyst. ACS Sustain. Chem. Eng..

[75] Choudhary V., Mushrif S.H., Ho C., Anderko A., Nikolakis V., Marinkovic N.S., Frenkel A.I., Sandler S.I., Vlachos D.G. (2013). Insights into the Interplay of Lewis and Brønsted Acid Catalysts in Glucose and Fructose Conversion to 5-(Hydroxymethyl)furfural and Levulinic Acid in Aqueous Media. J. Am. Chem. Soc..

[76] Zuo Y., Zhang Y., Fu Y. (2014). Catalytic Conversion of Cellulose into Levulinic Acid by a Sulfonated Chloromethyl Polystyrene Solid Acid Catalyst. ChemCatChem.

[77] Yang G., Pidko E.A., Hensen E.J. (2012). Mechanism of Brønsted acid-catalyzed conversion of carbohydrates. J. Catal..

[78] Fachri B.A., Abdilla R.M., Bovenkamp H.H.v.d., Rasrendra C.B., Heeres H.J. (2015). Experimental and Kinetic Modeling Studies on the Sulfuric Acid Catalyzed Conversion of d-Fructose to 5-Hydroxymethylfurfural and Levulinic Acid in Water. ACS Sustain. Chem. Eng..

[79] Bevilaqua D.B., Rambo M.K., Rizzetti T.M., Cardoso A.L., Martins A.F. (2013). Cleaner production: levulinic acid from rice husks. J. Clean. Prod..

[80] Wettstein S.G., Alonso D.M., Chong Y., Dumesic J.A. (2012). Production of levulinic acid and gamma-valerolactone (GVL) from cellulose using GVL as a solvent in biphasic systems. Energy Environ. Sci..

[81] Khan A.S., Man Z., Bustam M.A., Kait C.F., Nasrullah A., Ullah Z., Sarwono A., Ahamd P., Muhammad N. (2018). Dicationic ionic liquids as sustainable approach for direct conversion of cellulose to levulinic acid. J. Clean. Prod..

[82] Ramli N.A.S., Amin N.A.S. (2015). A new functionalized ionic liquid for efficient glucose conversion to 5-hydroxymethyl furfural and levulinic acid. J. Mol. Catal. Chem..

[83] Jow J., Rorrer G.L., Hawley M.C., Lamport D.T. (1987). Dehydration of d-fructose to levulinic acid over LZY zeolite catalyst. Biomass.

[84] Ya’aini N., Amin N.A.S., Asmadi M. (2012). Optimization of levulinic acid from lignocellulosic biomass using a new hybrid catalyst. Bioresour. Technol..

[85] Yang H., Wang L., Jia L., Qiu C., Pang Q., Pan X. (2014). Selective Decomposition of Cellulose into Glucose and Levulinic Acid over Fe-Resin Catalyst in NaCl Solution under Hydrothermal Conditions. Ind. Eng. Chem. Res..

[86] Hayashi E., Yamaguchi Y., Kamata K., Tsunoda N., Kumagai Y., Oba F., Hara M. (2019). Effect of MnO_2_ Crystal Structure on Aerobic Oxidation of 5-Hydroxymethylfurfural to 2,5-Furandicarboxylic Acid. J. Am. Chem. Soc..

[87] Yi G., Teong S.P., Zhang Y. (2015). The direct conversion of sugars into 2, 5-furandicarboxylic acid in a triphasic system. ChemSusChem.

[88] Rahaman M.S., Tulaphol S., Hossain M.A., Jasinski J.B., Sun N., George A., Simmons B.A., Maihom T., Crocker M., Sathitsuksanoh N. (2022). Cooperative Brønsted-Lewis acid sites created by phosphotungstic acid encapsulated metal–organic frameworks for selective glucose conversion to 5-hydroxymethylfurfural. Fuel.

[89] Aranha D.J., Gogate P.R. (2023). A Review on Green and Efficient Synthesis of 5-Hydroxymethylfurfural (HMF) and 2, 5-Furandicarboxylic Acid (FDCA) from Sustainable Biomass. Ind. Eng. Chem. Res..

[90] Ricciardi L., Verboom W., Lange J.-P., Huskens J. (2022). Production of furans from C 5 and C 6 sugars in the presence of polar organic solvents. Sustain. Energy Fuels.

[91] Zhang Y., Cao Y., Yan C., Liu W., Chen Y., Guan W., Wang F., Liu Y., Huo P. (2023). Rationally designed Au-ZrOx interaction for boosting 5-hydroxymethylfurfural oxidation. Chem. Eng. J..

[92] Cheng X., Li S., Liu S., Xin Y., Yang J., Chen B., Liu H. (2022). Highly efficient catalytic oxidation of 5-hydroxymethylfurfural to 2,5-furandicarboxylic acid using bimetallic Pt–Cu alloy nanoparticles as catalysts. Chem. Commun..

[93] Rathod P.V., Jadhav V.H. (2018). Efficient Method for Synthesis of 2,5-Furandicarboxylic Acid from 5-Hydroxymethylfurfural and Fructose Using Pd/CC Catalyst under Aqueous Conditions. ACS Sustain. Chem. Eng..

[94] Megías-Sayago C., Lolli A., Ivanova S., Albonetti S., Cavani F., Odriozola J.A. (2019). Au/Al_2_O_3_ – Efficient catalyst for 5-hydroxymethylfurfural oxidation to 2,5-furandicarboxylic acid. Catal. Today.

[95] Pichler C.M., Al-Shaal M.G., Gu D., Joshi H., Ciptonugroho W., Schüth F. (2018). Ruthenium Supported on High-Surface-Area Zirconia as an Efficient Catalyst for the Base-Free Oxidation of 5-Hydroxymethylfurfural to 2,5-Furandicarboxylic Acid. ChemSusChem.

[96] Liu X., Zhang M., Li Z. (2020). CoOx-MC (MC = Mesoporous Carbon) for Highly Efficient Oxidation of 5-Hydroxymethylfurfural (5-HMF) to 2,5-Furandicarboxylic Acid (FDCA). ACS Sustain. Chem. Eng..

[97] Zhou H., Xu H., Liu Y. (2019). Aerobic oxidation of 5-hydroxymethylfurfural to 2, 5-furandicarboxylic acid over Co/Mn-lignin coordination complexes-derived catalysts. Appl. Catal. B.

[98] Chen G., Wu L., Fan H., Li B.-G. (2018). Highly efficient two-step synthesis of 2, 5-furandicarboxylic acid from fructose without 5-hydroxymethylfurfural (HMF) separation: *in situ* oxidation of HMF in alkaline aqueous H2O/DMSO mixed solvent under mild conditions. Ind. Eng. Chem. Res..

[99] Megías-Sayago C., Ivanova S., López-Cartes C., Centeno M.A., Odriozola J.A. (2017). Gold catalysts screening in base-free aerobic oxidation of glucose to gluconic acid. Catal. Today.

[100] Jin X., Zhao M., Vora M., Shen J., Zeng C., Yan W., Thapa P.S., Subramaniam B., Chaudhari R.V. (2016). Synergistic Effects of Bimetallic PtPd/TiO_2_ Nanocatalysts in Oxidation of Glucose to Glucaric Acid: Structure Dependent Activity and Selectivity. Ind. Eng. Chem. Res..

[101] Guo S., Fang Q., Li Z., Zhang J., Zhang J., Li G. (2019). Efficient base-free direct oxidation of glucose to gluconic acid over TiO_2_-supported gold clusters. Nanoscale.

[102] Cao Y., Liu X., Iqbal S., Miedziak P.J., Edwards J.K., Armstrong R.D., Morgan D.J., Wang J., Hutchings G.J. (2016). Base-free oxidation of glucose to gluconic acid using supported gold catalysts. Catal. Sci. Technol..

[103] An D., Ye A., Deng W., Zhang Q., Wang Y. (2012). Selective Conversion of Cellobiose and Cellulose into Gluconic Acid in Water in the Presence of Oxygen, Catalyzed by Polyoxometalate-Supported Gold Nanoparticles. Chem. Eur J..

[104] Qi P., Chen S., Chen J., Zheng J., Zheng X., Yuan Y. (2015). Catalysis and Reactivation of Ordered Mesoporous Carbon-Supported Gold Nanoparticles for the Base-Free Oxidation of Glucose to Gluconic Acid. ACS Catal..

[105] Lee J., Saha B., Vlachos D.G. (2016). Pt catalysts for efficient aerobic oxidation of glucose to glucaric acid in water. Green Chem..

[106] Jin X., Zhao M., Shen J., Yan W., He L., Thapa P.S., Ren S., Subramaniam B., Chaudhari R.V. (2015). Exceptional performance of bimetallic Pt_1_Cu_3_/TiO_2_ nanocatalysts for oxidation of gluconic acid and glucose with O_2_ to glucaric acid. J. Catal..

